# Taiwan Y-chromosomal DNA variation and its relationship with Island Southeast Asia

**DOI:** 10.1186/1471-2156-15-77

**Published:** 2014-06-26

**Authors:** Jean A Trejaut, Estella S Poloni, Ju-Chen Yen, Ying-Hui Lai, Jun-Hun Loo, Chien-Liang Lee, Chun-Lin He, Marie Lin

**Affiliations:** 1 Mackay Memorial Hospital, Taipei, Molecular Anthropology Laboratory, 45 Min-Sheng Road,225115 Tamsui, New Taipei city, Taiwan; 2 Laboratory of Anthropology, Genetics and Peopling History, Anthropology Unit, Department of Genetics and Evolution, University of Geneva, Geneva, Switzerland

**Keywords:** Y chromosome, Y-STR, Y-SNP, Austronesian migration, Taiwan, Island Southeast Asia, Haplogroup O1a

## Abstract

**Background:**

Much of the data resolution of the haploid non-recombining Y chromosome (NRY) haplogroup O in East Asia are still rudimentary and could be an explanatory factor for current debates on the settlement history of Island Southeast Asia (ISEA). Here, 81 slowly evolving markers (mostly SNPs) and 17 Y-chromosomal short tandem repeats were used to achieve higher level molecular resolution. Our aim is to investigate if the distribution of NRY DNA variation in Taiwan and ISEA is consistent with a single pre-Neolithic expansion scenario from Southeast China to all ISEA, or if it better fits an expansion model from Taiwan (the OOT model), or whether a more complex history of settlement and dispersals throughout ISEA should be envisioned.

**Results:**

We examined DNA samples from 1658 individuals from Vietnam, Thailand, Fujian, Taiwan (Han, plain tribes and 14 indigenous groups), the Philippines and Indonesia. While haplogroups O1a*-M119, O1a1*-P203, O1a2-M50 and O3a2-P201 follow a decreasing cline from Taiwan towards Western Indonesia, O2a1-M95/M88, O3a*-M324, O3a1c-IMS-JST002611 and O3a2c1a-M133 decline northward from Western Indonesia towards Taiwan. Compared to the Taiwan plain tribe minority groups the Taiwanese Austronesian speaking groups show little genetic paternal contribution from Han. They are also characterized by low Y-chromosome diversity, thus testifying for fast drift in these populations. However, in contrast to data provided from other regions of the genome, Y-chromosome gene diversity in Taiwan mountain tribes significantly increases from North to South.

**Conclusion:**

The geographic distribution and the diversity accumulated in the O1a*-M119, O1a1*-P203, O1a2-M50 and O3a2-P201 haplogroups on one hand, and in the O2a1-M95/M88, O3a*-M324, O3a1c-IMS-JST002611 and O3a2c1a-M133 haplogroups on the other, support a pincer model of dispersals and gene flow from the mainland to the islands which likely started during the late upper Paleolithic, 18,000 to 15,000 years ago. The branches of the pincer contributed separately to the paternal gene pool of the Philippines and conjointly to the gene pools of Madagascar and the Solomon Islands. The North to South increase in diversity found for Taiwanese Austronesian speaking groups contrasts with observations based on mitochondrial DNA, thus hinting to a differentiated demographic history of men and women in these populations.

## Background

The Taiwanese population comprises three groups of inhabitants. Taiwan Han (TwHan), presently the most numerous group, are descendants mainly of immigrants who came to Taiwan ~400 years ago and include Minnan and Hakka, as well as Han Chinese who immigrated more recently from all over China. The Taiwan mountain tribes Aborigines (TwMtA) include 12 of the 14 officially recognized ethnic groups of the island and represent about 2% of its total population. Finally, a minority group generally described as the Taiwan plain tribe Aborigines (TwPlt) or Pingpu, who are believed to be heavily sinicized and mixed with the Taiwanese Han [1]. This last group represents less than 1% of the population in Taiwan.

TwMtA, Filipinos and Indonesian people speak languages that belong to the Austronesian languages family [2]. Ten primary branches are usually recognized at the roots of this linguistic family [3]. Nine of these branches are found exclusively in Taiwan and constitute the first-order subgroup of the Austronesian language family. Austronesian languages spoken outside Taiwan, including the Taiwan offshore Yami language, belong to the tenth branch, Malayo-Polynesian [3]. This branch comprises more than 1,200 separate languages that are spoken over a huge geographic region covering Madagascar in the Indian Ocean, Island Southeast Asia (ISEA) and hundreds of Oceanic islands all the way east towards Easter Island in the Pacific. Thus, because of the particular geographic distribution of the first-order subgroups of Austronesian, Taiwan is often considered as the potential original homeland of Austronesian speakers.

In addition to linguistics, scholars have also examined evidence from archaeology and genetics to determine the original homeland of Austronesian speakers. The most consistent and generally accepted view based on archaeological evidence [4] suggests that Proto-Austronesian speakers, the ancestors of present-day Austronesian populations, reached ISEA via Taiwan, some 4,500 years ago, before the Iron Age, and possibly concomitant with the migration of early farming communities who expanded in Southeast China as a result of favorable climate changes [5].

Recent advances in molecular genetics using human mitochondrial DNA (mtDNA) complete genome sequencing, and genotyping of the haploid non-recombining Y chromosome (NRY) have generated a large amount of genetic data from populations in continental Asia and ISEA [6-14]. Using mtDNA from diverse genetic pools, Melton et al. [15] were first to support the idea that all present-day Austronesian speakers from Taiwan, ISEA and the Pacific region can retrace their origins with roots in Southeast Asia, via Taiwan (4). Later, other geneticists corroborated that the maternal genetic structure of the Austronesian speakers of Taiwan and of western ISEA (the Philippines and Western Indonesia) showed some remarkable similarities [16-19], an observation also echoed by linguistic evidence [20]. Such genetic results stimulated much debate when trying to correlate scenarios of past dispersal routes inferred independently by phylogeography, archeology and linguistics [21,22]. To reduce the controversy and possible contradictions between the various hypotheses, such as the express train and slow boat models to name a few [5,23], a more lenient description of the migration of the Austronesian peoples was later redefined as the “Out of Taiwan” (OOT) hypothesis [24]. Here the OOT identifies a unique movement of people leaving southeastern Taiwan more than 4,000 years ago and moving toward the Pacific and later to the Indian Ocean. Differences of opinion developed when Soares and colleagues [17], using mtDNA haplogroup E data, showed that it was also possible that people moved northward from ISEA toward Taiwan. This was soon supported by additional studies [18,25] both of which inferred a bidirectional genetic corridor between Taiwan and the Philippines, as well as others that showed possible simultaneous northern and southern passages to Taiwan and to Indonesia [14,26,27].

Despite all contentions, the use of maternally transmitted genetic markers from mtDNA and paternally transmitted markers such as single nucleotide polymorphisms (Y-SNPs) and microsatellites (Y-STRs) of the non-recombining portion of the Y chromosome (NRY) have now become powerful tools to describe independently distinct genetic patterns within and between populations and to retrace their movements across regions. These methods have not only become complementary to each other, but because of the lack of recombination, they are also very informative in tracing human prehistory, temporally and spatially [6].

Several studies have retraced the earliest expansion Y-chromosome macro-haplogroup O1-M175 in East Asia and ISEA [28-31]. It has now been shown that many Y-STR lineages defined in the background of O1-M175 and seen among TwMtA and ISEA populations derive their ancestry independently from Daic-speaking groups as a result of a pre-Neolithic demographic expansion from Southeast China [11,27,30]. It has also been shown that not all paternal lineages observed among the populations of ISEA trace back their origin to Taiwan, but instead that TwMtA and ISEA populations find a common origin in Southeast China [7,14,17,26,27,30,32,33]. Nevertheless, the phylogenetic resolution of haplogroup O chromosomes in East Asia populations in general and in Taiwanese samples in particular, still remains generally rudimentary. Here, 81 high-definition Y-chromosome SNPs [8] and 17 microsatellites [32-34] are examined in combination for the first time to determine the genetic profiles of extant populations of Taiwan, the Philippines, Indonesia, and populations from the Indochinese peninsula in Mainland Southeast Asia (MSEA, referred herein as Indochina), namely Thailand, North Vietnam and the Akha. This investigation was undertaken with the aim of evaluating the distribution pattern of male-specific diversity in Taiwan, the Philippines and Indonesia and to see if this is consistent with a single pre-Neolithic expansion scenario from Southeast China to all ISEA, or if it better fits the OOT model, or whether a more complex history of settlement and dispersals throughout ISEA should be envisioned.

Our results support a northeastward dispersal from Southeast Asia (SEA) to Taiwan and Island Southeast Asia and corroborate the hypothesis of separate migration routes to Indonesia from SEA and Indochina [14,18,25-27] (i.e.. a northeastward dispersal from Southeast Asia (SEA) via Taiwan to Island Southeast Asia, and a southward dispersal via the Indochinese peninsula to ISEA. We extend earlier observations by proposing a pincer model that includes a multidirectional center of dispersal in SEA, contiguous northward migration routes from Indonesia to the Philippines and Taiwan and southward gene flow from Taiwan through the Philippines and Indonesia. Finally, the genetic structure of Taiwan plain tribe Aborigines may introduce new speculation on the time of their first settlement in Taiwan.

## Methods

### Samples

**Figure 1:**
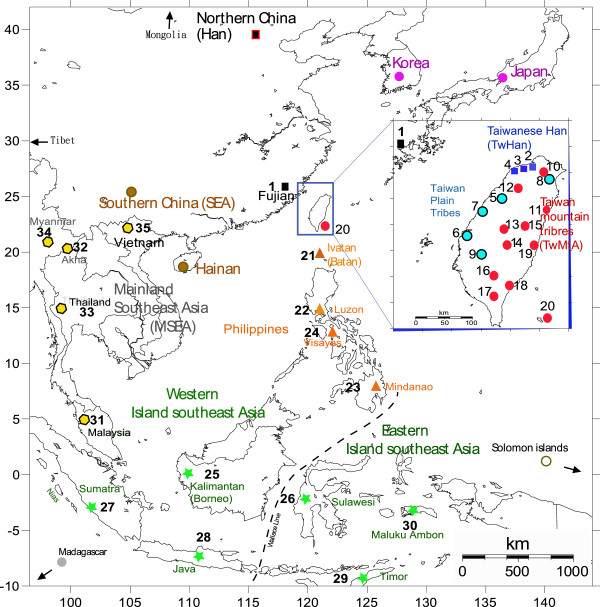
**Geographic map showing the locations of the samples of Fujian, Taiwan, the Philippines and Indonesia genotyped in this study.** Group numbers identifying sampling locations are described in Table 1, and color codes are : Black squares = Mainland Han, Dark blue squares = Taiwan Han, Sky blue circles = Taiwan Plain tribes, Red circles = Taiwan Mountain tribe Aborigines, Orange triangles = Philippines, Green stars = Indonesia, Yellow hexagons = Indochina (Indochina). The Wallace’s line (black dotted line) separates Eastern ISEA from Western ISEA. Locations without a number indicate groups obtained from additional literature (Additional file 1: Table S1), namely: Brown circle = Yueh/Daic/Hainan in south China, Grey = Madagascar, White = Solomon island, Pink = Japan and Korea, and Black with red frame = North China.

Whole blood or saliva specimens were collected from 1660 unrelated male individuals from 35 ethnic groups in ISEA, Taiwan, the east coast of China and Indochina (Vietnam and Thailand) (Table 1 and Figure 1).These samples (Figure 1) comprise Austronesian speaking groups from the Philippines (n = 146) and Indonesia (n = 246), and from eleven TwMtA populations (n = 355). Among the latter, Atayal, Truku (Taroko) and Saisiyat and Thao are all considered as northern Mountain Tribe Aborigines (n = 112), Rukai, Paiwan, Puyuma, Amis and the offshore Yami are all considered as southern Mountain Tribe Aborigines (n = 146), whereas the Thao, Tsou and Bunun are referred here as central mountain tribes Aborigines. The sampling also includes Han groups from Taiwan, namely TwHAN comprising Minnan (n = 60), Hakka (n = 34), and a group composed of miscellaneous Minnan individuals (n = 258) who were uncertain about the origin of at least one of their parents (referred herein as MiscHan), as well as a sample of Han from Fujian (n = 55), facing Taiwan on the eastern coast of China. Finally groups from Indochina, namely Vietnam (Hanoi, n = 24), Thailand (Bangkok n = 35 and Akha n = 27) and Malaysia (n = 8) were included to represent information on the Indochinese peninsula.

**Table 1:** Information on the 35 population samples genotyped in the present study

**Sample number**^**1**^	**Country**	**Ethnicity**	**ISO639-3 (or local) abbreviation**^**2**^	**Linguistic family**	**Language branch**	**Population**	**Province/City/Region**	**County**	**Sample size**
1	China (Han)	Fujian	nan	Sino-Tibetan	Hokien (Minnan)	34.880,000	Southeast Coast	Fujian	55
2	Taiwan (Han)	Miscellaneous Han (including undefined Minnan/Hakka)	ad	Sino-Tibetan	Hokien (Minnan)	14,000,000	Taiwan	Taipei	258
3		Minnan	nan	Sino-Tibetan	Hokien (Minnan)	14,000,000	Taiwan	Taipei	60
4		Hakka	hak	Sino-Tibetan	Hakka	2,370,000	Taiwan	Xinzhu	34
5	Taiwan Plain tribes/Pingpu	Kulon-Pazeh	uun	Sino-Tibetan	Hokien (Minnan)	300	Taiwan West Coast	Puli	40
6		Yunlin*	fos	Sino-Tibetan	Hokien (Minnan)	10,000	Taiwan West Coast	Yunlin	21
7		Papora*	ppu	Sino-Tibetan	Hokien (Minnan)	-	Taiwan West Coast	Gomach	18
8		Ketagalan	kae	Austronesian	extinct Paiwanic	1,000	Northeast Taiwan	Yilan	30
9		Siraya	fos	Austronesian	Taivoa and Makatao	10,000	Southwest Taiwan	Southwest Taiwan	261
10	Taiwan (Mountain tribes Aborigines)	Atayal	tay	Austronesian	Atayalic	80,061	Moutain tribes	Wulai	52
11		Truku (Taroko)	trv	Austronesian	Atayalic	25,800	Moutain tribes	Hualien	20
12		Saisiyat	xsy	Austronesian	Northwest Formosan	5,900	Moutain tribes	Wofeng	24
13		Thao	ssf	Austronesian	Paiwanic	693	Moutain tribes	Nantou	16
14		Tsou	tsu	Austronesian	Tsouic	6,733	Moutain tribes	Jiaye	41
15		Bunun	bnn	Austronesian	Bunun	51,447	Moutain tribes	Nantou	56
16		Rukai	dru	Austronesian	Rukai	11,911	Moutain tribes	Kaohsiung	29
17		Paiwan	pwn	Austronesian	Paiwanic	88,323	Moutain tribes	Taitung	25
18		Puyuma	pyu	Austronesian	Paiwanic	11,850	Moutain tribes	Taitung	23
19		Amis	ami	Austronesian	East Formosan	183,799	East Coast	Hualien	39
20		Yami	tao	Austronesian	Malayo-Polynesian	3,748	Southeast island	Tao/LanYu	30
21	Philippines	Batan	ivb	Austronesian	Malayo-Polynesian	1,350	Philippines	Philippines	24
22		Luzon	lz	Austronesian	Malayo-Polynesian	47,000,000	Philippines	Philippines	61
		Luzon unknown							3
23		Mindanao	mind	Austronesian	Malayo-Polynesian	16,000,000	Philippines	Philippines	8
24		Visayas	vis	Austronesian	Malayo-Polynesian	6,398,628	Philippines	Philippines	31
		Philippines unknown							19
25	Indonesia	Borneo (Kalimantan)	bor	Austronesian	Malayo-Polynesian	11,331,558	Borneo	Indonesia	22
		Borneo unknown							3
26		Sulawesi	sul	Austronesian	Malayo-Polynesian	14,111,444	Sulawesi	Indonesia	17
27		Sumatra (South)	sum	Austronesian	Malayo-Polynesian	43,309,707	Sumatra	Indonesia	18
		Sumatra unknown							8
28		Java (Central, East or West)	jav	Austronesian	Malayo-Polynesian	75,200,000	Java	Indonesia	131
		Javanese unknown							10
29		Timor	tim	Austronesian	Central-Eastern Malayo-Polynesian	3,800,000	Timor	Indonesia	4
30		Maluku (Ambon)	mal	Austronesian	Central-Eastern Malayo-Polynesian	2,549,454	Maluku	Indonesia	18
		Nusa Tengarra							1
		Indonesian unknown							14
31	Malaysia	Peninsular Malaysia	zsm/meo	Austronesian	Malayo-Polynesian	2,600,000	North Perak	Taiping	8
32	Thailand	Akha	ahk	Sino-Tibetan	Akha	56,600	Changmai	Changmai	27
33		Thailand	tha	Tai-Kadai (Daic)	Tai	20,200,000	Bangkok	Bangkok	75
34	Myanmar	Union of Myanmar	mian	Sino-Tibetan	Burmic	28,877,000	Northeast Burma	Yonggong	2
35	Vietnam	Vietnam	vie	Austro-Asiatic	Mon-Khmer	65,800,000	Hanoi	Hanoi	24
Total									1660

^1^Sample numbers as in Figure 1.

^2^Detailed information and ISO639-3 codes can be searched online version of “Ethnologue” by: Lewis, M. Paul [35]: http://www.ethnologue.com/.

*Not clearly defined as plain tribes.

All individuals gave information on their familial birthplace, consented to participate in this project, and the study and the informed consent protocol was approved by the ethics committee of the Mackay Memorial Hospital in Taipei (Taiwan).

DNA was extracted from blood or saliva using QIAmp DNA kit (Qiagen inc. USA) and ORAgene DNA self-collection kit (DNA Genotek Inc. Canada) respectively, with minor modifications to the procedure recommended by the manufacturer.

Comparative data with other populations of south China, ISEA and Oceania were taken from the literature and comprised Yueh/Daic-speaking populations [27,30,36-38], Malayo-Polynesians and Papua New Guineans [39], and Han Chinese [7,8,12,29,30,40,41]. These datasets are shown in Additional file 1: Table S1.

### Genetic analysis

Using the PCR-SSP method, a combination of 81 markers, the majority of which are slowly evolving SNPs [8], were used to genotype in a hierarchical fashion the NRY of 1660 individuals. Altogether, these 81 markers define 68 Y-SNP haplogroups: CDEF-M168, C*-M216, C1-M8, C2-M38, C3-M217, C4-M247, C5-M356, C6-P55, DE-M145, D*-M174, D1-M15, E-M96, F*-M89, G-M201, , H-M69, H1-M52, H1a-M82, H2-Apt, I-M170, J-P209, K-M9, K1-M147, L-M20, M-P256, NO-M214, N-M231, N1-LLY22g, N1a-M128, N1c*-Tat, N1c1-M178, O-M175, O1a*-M119, O1a1*-P203, O1a1a-M101, O1a2-M50, O2*-P31, O2a*-PK4, O2a1*-M95, O2a1a-M88, O2b-SRY465, O3*M122, O3a*-M324, O3a1*-KL1, O3a1a-M121, O3a1b-M164, O3a1c*-IMS-JST002611, O3a1c1-P103, O3a2*-P201, O3a2a-M159, O3a2b*-M7, O3a2b1-M113, O3a2c*-P164, O3a2c1*-M134, O3a2c1a-M133, O3a2c1a1-M162, O3a2c1b-P101, O3a3-M300, O3a4-M333, PQR-M45, Q-M242, R*-M207, R1-M173, R1a1*-SRY10831.2, R1a1a-M17, R1b-M343, R2a-M124, S-M230, and T1-M70. Chromosomes were assigned to haplogroups according to the improved methods of Karafet et al. and Yan et al. [8,42], and to the classification provided by International Society of Genetic Genealogy for the Y Chromosome Consortium (YCC) [43,44]. In this study, microsatellite MSY2.2, usually used to differentiate haplogroup O* from O1*, was not used on all samples; accordingly the two haplogroups were treated conjointly as O*/O1* and are referenced as O*/O1*-M175 (xM119, P31, M122) in the analyses.

PCR was performed in 10 μl reactions containing 0.5 U AmpliTaq Gold polymerase (Applied Biosystems), 10 mM Tris–HCl (pH 8.3), 50 mM KCl, 2 mM MgCl_2_, 0.1 mM each of the four deoxyribonucleotide triphosphates, 0.2 μM each of forward/reverse primers [45] and 50–100 ng genomic DNA. Thermal cycling conditions were 94°C for 10 min, and then 14 denaturing cycles at 94°C for 20 sec, primer annealing at 63–56°C using 0.5°C decrements and extension at 72°C for 1 min, followed by 20 cycles at 94°C for 20 sec, 56°C for 20 sec, 72°C for 1 min and a final 5-min extension at 72°C [46]. Prior to sequencing, excess dNTP and primers were removed from the PCR products by pre-treating with shrimp alkaline phosphatase (Sap) and Exonuclease I (*Exo I*) enzymes (USB Product number US 70995 pre-sequencing Kit, Pharmacia) following the conditions recommended by the manufacturer (37°C for 30 min and 80°C for 15 min respectively). Sequencing was performed on both strands using the DyeDeoxy Terminator Cycle Sequencing Kit (Applied Biosystems) according to manufacturer recommendations. Purification on a G50 sephadex column was performed before the final run on an automated DNA sequencer (ABI Model 377).

All samples were further subjected to genotyping with 17 microsatellites (DYS19, DYS385I, DYS385II, DYS389I, DYS389II, DYSS390, DYS391, DYS392, DYS393, DYS437, DYS438, DYS439, DYS448, DYS456, DYS458, DYS635, and Y GATA-H4) and analyzed using Y-filer kit (Applied Biosystems). In brief, PCR products were mixed with GeneScan 500LIZ (Applied Biosystems) as internal size standard and analyzed by capillary electrophoresis with an ABI Prism 310 genetic analyzer (Applied Biosystems) in the mode of standard fragment analysis protocol. Genetyper 2.5.2 (Applied Biosystems) was used for allele scoring. For all statistical and network analyses, we used data from DYS389II by subtracting DYS389I from DYS389II [46].

### Statistical and population genetic analysis

The Y-chomosome SNP dataset was used to obtain frequency distributions of haplogroups (clades and sub-clades) in the population samples by mere counting, and the unbiased gene diversity index, *h*, and its standard error were calculated using the formulas given by Nei [47].

Contour maps of interpolated spatial frequency variations of the most relevant clades in East Asia were constructed by applying the Kriging algorithm in Surfer 8.0 (Golden software). Similarly, the internal diversity of each relevant Y-SNP clade was estimated on the basis of its STR variation by computing the average variance in repeat size over STR loci (the rho statistic), following the method of Zhivotovsky et al. [48], and the spatial variation of this statistic was also interpolated with Surfer 8.0.

Thirteen Y-STR loci (DYS19, DYS389 I/II, DYS390, DYS391, DYS392, DYS393, DYS385a/b, DYS437, DY438, DYS439, DYS635 and Y GATA-H4) were used to estimate the age of the variation within each SNP haplogroup following the modified coalescence method of Zhivotovsky et al. [48-50], assuming a generation time of 25 years and a single mutation rate of 0.00069 per locus per generation. However, the results were very similar to those obtained only with the seven most commonly used STRs in the data retrieved from the literature. Accordingly, the same seven Y-STR loci, or only five of them in the worst cases (noted in italic here after), were used to accommodate the literature data (DYS19, *DYS389 I*, DYS389 II, *DYS390, DYS391, DYS392*, and *DYS393*). Age estimates of STR variation of haplogroups comprised less than 10 individuals were also calculated but results are to be considered with caution.

Gene contribution estimates between populations were inferred by two methods; firstly the coalescent approach of Admix version 2.0, which infers contributions from parental populations according to STR haplotype frequencies considering each haplotype as an allele of the same locus [51,52], and secondly using the analysis of shared STR lineages (LS) between populations [53] to infer those contributions as well as to determine the unshared gene portion in the hybrid population. The Fujian and Taiwan Han samples were pooled so as to constitute a putative Han parental contributor and a pool of most samples of Austronesian speaking groups, namely TwMtA, Filipinos and Western Indonesians (i.e. all samples from Borneo, Sumatra and Java) as the other putative parental contributor. Seven STRs were used in these analyses (i.e. DYS19, DYS389I, DYS389II, DYS390, DYS391, DYS392 and DYS393.

Both the SNP and STR datasets were used to perform AMOVA analyses of population structure using the Arlequin 3.5.1.2 software [54]. Multidimensional scaling (MDS) analysis was performed to represent patterns of genetic relationships between all groups in our data set. A Reynolds distance matrix was obtained from frequency distributions of SNP haplogroups with Arlequin, and used as input for MDS analysis. MDS plots were constructed using XLSTAT software version 7.5.2 [55].

Median joining (MJ) networks of Y-STR haplotypes (defined by DYS19, DYS389I, DYS389II, DYS390, DYS391, DYS392 and DYS393) for relevant SNP haplogroups and sub-haplogroups of the O clade were constructed from reduced median joining networks using the NETWORK 4.1.0.6 software [56]. We used a reduction of one and locus specific weights based on the relative mutation rates of the following Y-STR loci: DYS19 (weight of 5), DYS389I (4), DYS389II (3), DYS390 (2), DYS391 (2), DYS392 (20), and DYS393 (20) [57-60].

## Results

### Y chromosome diversity and its geographic distribution

**Figure 2:**
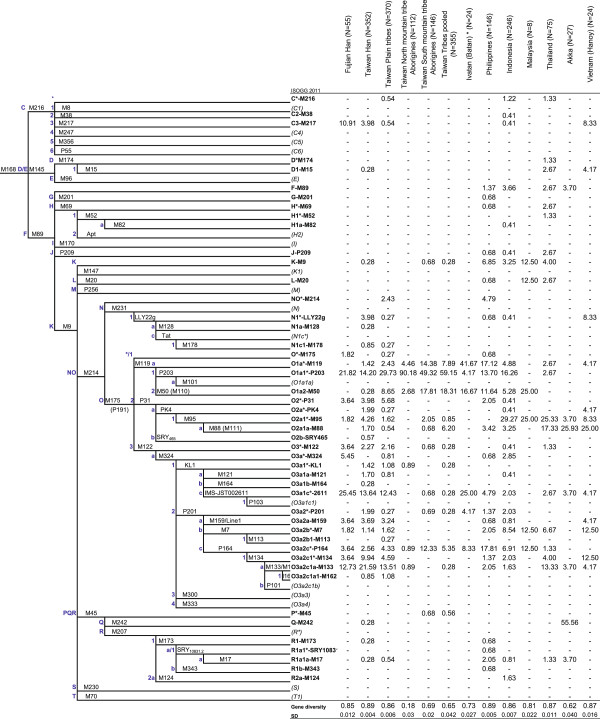
**Phylogenetic tree of 47 Y-chromosome haplogroups seen in this study (shown in boldface) and hierarchically defined using 81 slowly evolving binary markers (68 in the Figure).** The marker names are shown along the branches, and haplogroup names are shown on the right side according to ISOGG Y-DNA Haplogroup Tree 2011. Potentially paraphyletic undefined subgroups are distinguished from recognized haplogroups by the asterisk symbol. Haplogroups tested for but not seen in this study are shown in (italic). See Additional file 1: Table S2 for a more detailed frequency table.

**Figure 3:**
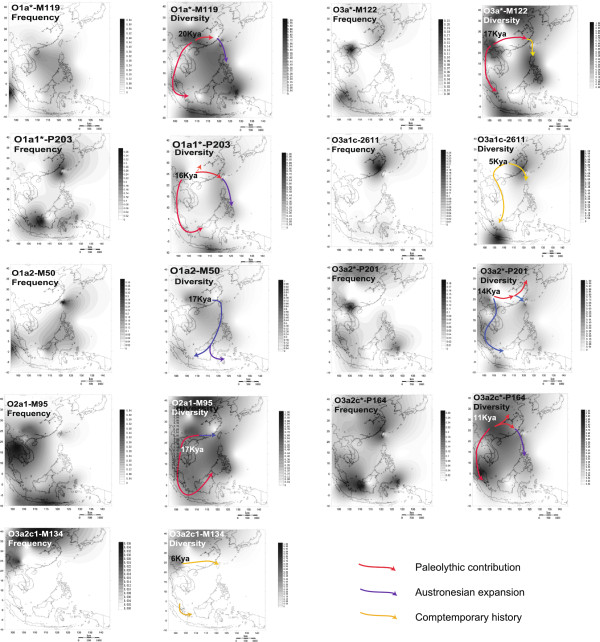
**Spatial distributions of the O1, O2 and O3 clades using haplogroup frequency and associated STR diversity (rho statistic).** Maps are based on data from Additional file 1: Tables S1 and S2 and from literature data [12,27,30,36,39,40,62]. Panels are labeled according to ISOGG2011 [8,42,43]. Arrows symbolize dispersals and gene flow, and stages (B, C and D) are according to Karafet et al. [26] (see Discussion section). The age at the beginning of arrows represents the likely time of origin of the haplogroup as estimated from its STR diversity (Table 2 and Additional file 1: Table S3), and arrow colors represent time of dispersal (red for Paleolithic, blue for Neolithic/Austronesian expansion and yellow for contemporary historical times).

**Figure 4:**
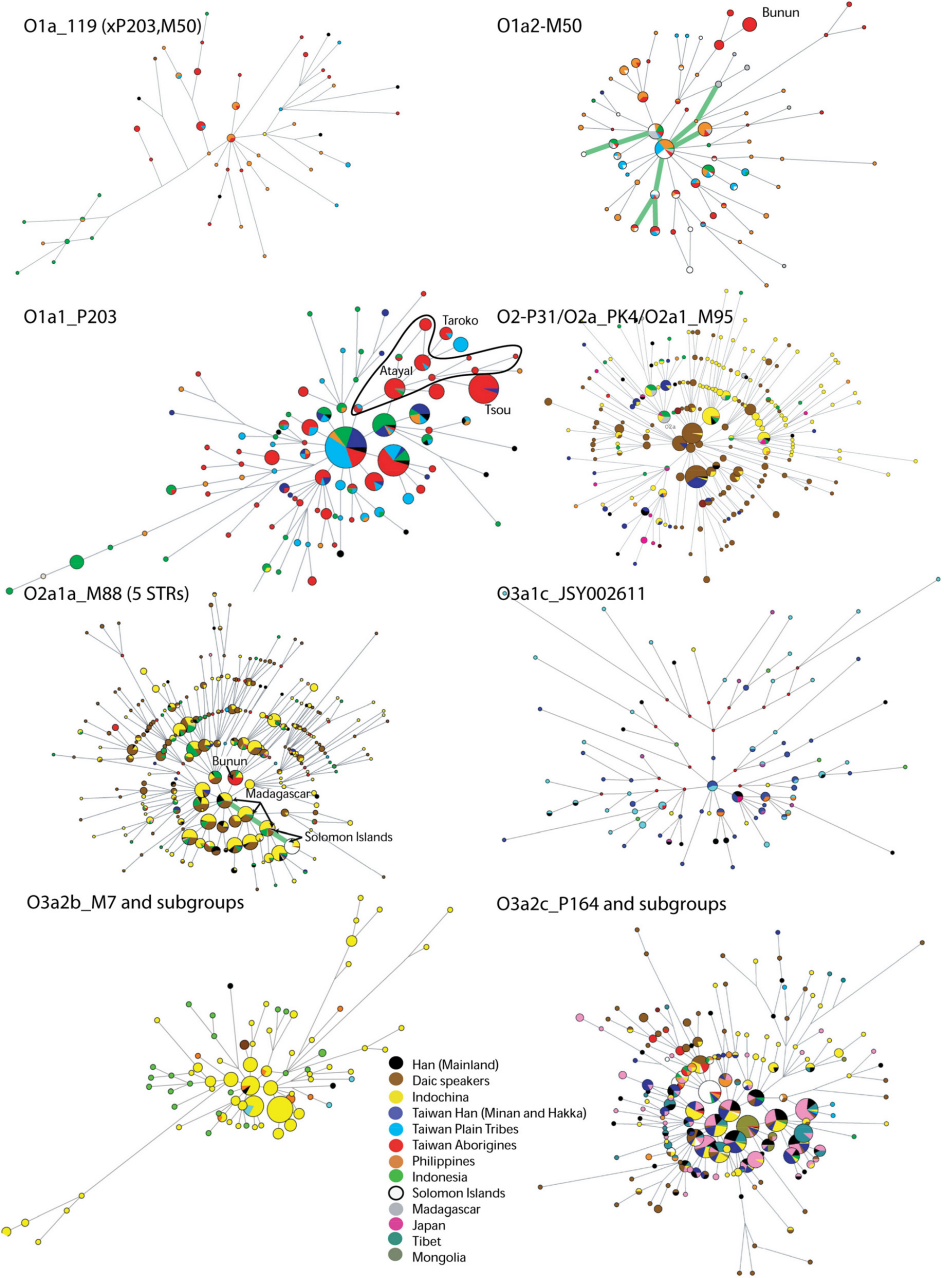
**Median joining Networks for the whole dataset and published East Asian Y haplogroup lineages based on seven Y-STR loci **[7,8,12,29,30,41]**.** Sizes of circles are proportional to Y-STR haplotype frequency; lines between circles (links) represent mutation differences. Green links in O1a2 and O2a1a represent ancestry sharing between Solomon and Madagascar (note that only TwMtA O1a2 is included in this pathway. Colors within circles indicate populations.

The frequency distribution of Y-chromosome SNP haplogroups detected in Taiwan, ISEA and Indochina is shown in Figure 2 and reported in detail in Additional file 1: Table S2, and are summarized is Additional file 1: Figures S1 and S2. Additional file 1: Figure S3 displays the variation of diversity measures according to latitude among TwMtA. The interpolation contour maps resulting from applying the Kriging method both to the frequency distributions of Y-SNP clades and their internal STR diversity are shown side-by-side in Figure 3. Forty seven out of the 81 genotyped Y-SNPs were observed in the derived state [61], thus defining 47 haplogroups observed in our samples, that belong to major clades C, D, F*, G, H, J, K*, L, N, O, P*, Q and R. MJ networks of major Y-SNP clades in SEA are shown in Figure 4. Age estimates of the diversity of these clades, based on the assumption that mutations in haplotypes accumulate in situ (i.e. no gene flow), are reported in Table 2 and Additional file 1: Table S3.

**Table 2:** Age estimates of the diversity accumulated in Y-haplogroups using a 7 Y-STRs dataset

	**Age in Kya (n)**^**2**^						
	**± SE**						
**Haplogroup**^**1**^	**Taiwan Han**^**3**^	**Plain tribes**	**Taiwan mountain tribes**	**Philippines**		**Western Indonesia**	**All populations**
				**Batan only**	**Philippines only**		
O1a*-M119	16.56 (5)	13.38 (7)	19.96 (28)	10.82 (10)	20.36 (15)	4.14 (5)	24.67 (80)
	± 4.39	± 2.575	± 5.53	± 2.85	± 5.65	± 2.67	± 5.31
O1a1*-P203	8.59 (50)	10.74 (93)	16.29 (210)	9.83 (10)	16.05 (15)	7.28 (32)	13.45 (437)
	± 3.27	± 6.6	± 5.88	± 3.51	± 3.57	± 4.00	± 4.90
**O1a2-M50**	na^4^	8.63 (30)	17.54 (67)	na	12.48 (16)	7.06 (11)	14.23 (128)
		± 3.18	± 3.56		± 5.95	± 2.63	± 3.73
O2a1*-M95	10.35 (15)	8.63 (30)	8.05 (9)	na	14.73 (13)	14.74 (59)	14.23 (128)
	± 2.28	± 3.18	± 2.32		± 7.64	± 2.86	± 3.73
O3a1c*-IMS-JST002611	19,41 (48)	16,51 (42)	na	0.86 (6)	na	28.47 (4)	14.23 (128)
	± 6.62	± 4.74		± 0.86		± 13.76	± 3.73
O3a2c* M164	19.55 (9)	10.35 (13)	13.62 (19)	na	13.37 (24)	6.90 (14)	13.56 (65)
	± 7.73	± 3.48	± 5.12		± 2.82	± 2.10	± 3.59
**O3a2c1a-M133**	12.40 (38)	8.70 (44)	8.63 (6)	na	na	na	11.40 (94)
	± 3.18	± 3.19	± 4.13				± 5.11

^1^The seven Y-STRs used are: DYS19, DYS389 I, DYS389 II DYS390, DYS391, DYS392, and DYS393.

^2^Kya: thousands of years; ( n): sample size; SE : standard error.

^3^Taiwan Han include Minnan, Hakka and MiscHan.

^4^na: age was not estimated if less than 5 individuals bear the haplogroup.

The most prominent clade in Taiwan, O-M175 as a whole, represents almost 90% of Y chromosomes among the TwHan, about 95% among the TwPlt and more than 99% among the TwMtA (Additional file 1: Figures S1 and S2). Only one representative of the basal O*/O1*-M175 (xM119, P31, M122) was seen in each of the Luzon (Philippines), Fujian and TwPlt samples (Figure 2 and Additional file 1: Table S2). All other haplogroups of the O clade were observed at the derived state for the M119, P31 and M122 markers (Figure 2, Additional file 1: Table S2, and Additional file 1: Figures S1 and S2).

Haplogroup O1a*-M119 (Figure 2 and Additional file 1: Table S2) is seen throughout Batan (42%), the Philippines (4% to 33%) and Indonesia (4% to 18%). It has a patchy distribution among TwMtA (3-33%) and only some southern TwMtA show frequencies greater than 10% (i.e. Puyuma, Paiwan and Yami). O1a*-M119 was not observed in our Amis sample, neither in the Bunun, Saisiyat and Thao and has a low frequency among TwHan (1.4% to 2%). O1a*-M119 was neither observed outside Taiwan, among the Kalimantan in Borneo nor in Sumatra. However, the contour map interpolation (which includes published data, see Additional file 1: Table S1) indicates its presence in Western Sumatra, towards the Indian Ocean (Figure 3). The internal STR diversity of O1a*-M119 decreases gradually from mainland China towards the south, although a second, somewhat lower peak of diversity is observed around the Moluccas. The MJ network of O1a*-M119 (Figure 4) clearly differentiates TwMtA and TwPlt from Indonesia (Indonesian STR haplotypes are all included in the lower left reticulation of the O1a*-M119 network), whereas most Filipinos O1a*-M119 haplotypes are shared or very similar to those found among TwMtA and TwPlt. Age estimates based on the amount of molecular variation for O1a*-M119 were higher among TwMtA (19.96 ± 5.93 Kya) and the Philippines (20.36 ± 5.65 Kya) than in Indonesia (4.14 ± 2.67 Kya) (Table 2 and Additional file 1: Table S3).

O1a1*-P203, derived from O1a*-M119 (Figure 2 and Additional file 1: Table S2), is the most common haplogroup among the northern TwMtA and the Tsou (90%), while among southern TwMtA it represents about half of the Y chromosomes observed. It is also quite common in several TwPlt (e.g. Pazeh, Ketagalan and Siraya in various locations), but less frequent in the Philippines (13.7%) and Indonesia (16.3%), although it is observed in 36% of the Kalimantan in Borneo. It is also commonly seen in Han (22% and 14.2% in Fujian and TwHan, respectively), but uncommon in Thailand (2.7%), and was not observed in the Vietnamese (Hanoi) and the Akha. Accordingly, the contour map of the frequency variation of O1a1*-P203 shows two main locations of high frequency, in Taiwan and southwestern Borneo, and decreasing frequencies from these locations towards the Philippines, whereas the contour map of its internal diversity is more complex (Figure 3). The O1a1*-P203 MJ network has a marked star-like shape, with a central frequent haplotype detected throughout mainland and insular SEA (Figure 4). Interestingly, haplotypes observed in the Atayal branch off from this central node to form a distinct network, suggesting a founding event and a period of isolation in this population. The intriguing observation of Tsou O1a1*-P203 haplotypes deriving from those of Atayal evidenced by the seven STRs MJ network is no longer sustained when using more than 13 Y-STRs, as the two groups then split at an earlier founding level (13 Y-STR MJ network, data not shown). Age estimates of O1a1*-P203 in TwMtA (16.3 ± 5.9 Kya) and the Philippines (16.05 ± 3.6) were higher than estimates obtained for TwHan, Fujian or Western Indonesia (8.59 ± 3.3, 11.6 ± 6.1 and 7.28 ± 4.0, respectively) (Table 2 and Additional file 1: Table S3). Haplogroup O1a1a-M101 [46] is a para-group of O1a1*-P203 (Figure 2). It was tested on all O1a1*-P203 in our dataset but was not seen.

Haplogroup O1a2-M50 (or O1a2-M110, Figure 2 and Additional file 1: Table S2), a sister haplogroup of O1a1*-P203, is frequently observed among southern TwMtA (from 18% to 28%) and in the Bunun (61%), variable in the Philippines (from 3% to 16.7%), but rather rare in Western Indonesia (Java and Sumatra, ~5.3%). Note however that a high frequency of this haplotype has been reported in South Nias (~80%), an island of the Indian Ocean in front of Sumatra [26,63,64]. O1a2-M50 was not seen in our dataset in the Yami and was rare in TwHan, Fujian, Thailand, Vietnam and Malaysia. However, it has been reported as prevalent among a few Daic-speaking groups from southern China and Hainan (3% to 25%) [26,65], thus explaining the decrease in frequency and molecular variation of O1a2-M50 from Taiwan to the Philippines and Indonesia (Figure 3). Age estimates of diversity of this haplogroup decrease from Taiwan (17.54 ± 3.56 Kya) to the Philippines (12.5 ± 5.9 Kya) and Western Indonesia (7.06 ± 2.63 Kya) (Table 2 and Additional file 1: Table S3). The star-like shape of the O1a2-M50 MJ network places in the central nodes the STR haplotypes observed among TwMtA, TwPlt, Filipinos and Indonesians (Figure 4). We also notice that the haplotype continuity observed along the Taiwan-Philippines-Indonesian pathway can be traced further away toward Madagascar and the Solomon Islands [7,66,67].

Haplogroup O2 is comprised of two derived branches (Figure 2). The first branch includes subtypes O2*-P31 observed in Han (4%), TwPlt (5.7%), the Philippines (Luzon, 4.7%) and Indonesia (3.25%), and O2a*-PK4, O2a1*-M95 and O2a1a-M88 mostly seen among groups speaking Daic languages in South China and Indochina, and in Indonesia (Additional file 1: Table S2). The second O2 branch includes subtypes of O2b-SRY_465_ that are only found among Korean, Japanese and northern Chinese [11,68]. The distribution of the recently defined O2a*-PK4 [42] was reanalyzed by two separate laboratories (P. Underhill, Stanford University School of Medicine, CA, USA, personal communication and this study) using a total of 105 individuals (data not shown). The A to T transversion of PK4 [8,42] was confirmed to be ancestral to the M95 SNP (Figure 2). In our dataset, haplogroups O2a1-M95 and O2a1a-M88 occurred principally in Indochina (~20% and 20% for O2a1-M95 and O2a1a-M88, respectively, in Thailand, and 8% and 25% in Vietnam) and Indonesia (29% and 3%), and had a scattered distribution between Fujian, TwHan and TwPlt, with rather low frequencies. Among the TwMtA, the Yami (10% and 3.33% for O2a1-M95 and O2a1a-M88, respectively) and Bunun (0% and 37.5%) were the only Mountain tribes bearing haplogroup O2. While a study reported the presence of O2a1-M95 and O2a1a-M88 in Mindanao [7], only O2a1a-M88 (3%) was seen in our Philippines dataset (6.45% in Visayas, 3.42% on average for all the Philippines). We notice that the most frequent O2a1a-M88 Y-STR haplotype in the Bunun is also observed in China, among Daic-speaking populations, in Indochina and in Indonesia, but not among Solomon islanders, TwPlt or Han (Figure 4). In fact, Bunun O2a1a-M88 lineages do not belong to the MJ branch observed in the Solomon Islands or Madagascar.

Eleven O3 lineages, out of the 19 described by Karafet et al. [8,26,39] were observed in this study (Figure 2 and Additional file 1: Table S2). The most widely distributed sub-haplogroups of O3 in our dataset are O3a1c*-IMS-JST002611, O3a2*-P201, O3a2b*-M7 and O3a2c*-P164.

A rather high prevalence of haplogroup O3a1c*-IMS-JST002611 is observed in Fujian Han (~25%) and in the Taiwanese Minnan and Hakka (13% and 21%), as well as among the TwPlt (12%), whereas it occurs at an extremely low frequency in TwMtA (it was only observed in the Yami, at 3%), in the Philippines (0.8%) and in Indonesia (2%) (Figure 2 and Additional file 1: Table S2). Its high frequency among the Ivatan (25%) of the Batan archipelago north of the Philippines is however associated with a low age estimate (0.9 Kya) (Table 2 and Additional file 1: Table S3). The MJ network of this haplogroup is characterized by the presence of Han Y-chromosomes at most founding nodes (Figure 4). Accordingly, a location of high frequency of haplogroup O3a1c*-IMS-JST002611 emerges from the contour map, centered in the southeastern Chinese coast, with no clear clinal pattern of frequency variation across SEA (Figure 3). The contour map of O3a1c*-IMS-JST002611 diversity displays a high peak in Indonesia, but this is due to a few (only 4), molecularly distant STR-haplotypes which translates to an older but less precise age estimate (28.47 ± 13.76 Kya, Table 2 and Additional file 1: Table S3).

Other commonly observed haplogroups in the O3 series are the paragroups of the O3a2*-P201 branch (Figure 2 and Additional file 1: Table S2). Note that mutation P201 has not been tested in the Daic-speaking groups of China and Hainan [27], in which the resolution of O3* included O3*, O3a*, O3a1*, O3a1b, O3a1c, O3a2*, O3a2a, O3a2c*, O3a3 and O3a4 [42]. Here O3a2*-P201 did not exceed 2% in any pooled population group of our data set, and reached 4.35% in the Puyuma. These low frequencies and the high age estimates seen in Taiwan (i.e. Puyuma) (15 ± 3 Kya) and Western Indonesia (17 Kya ± 2 Kya; Additional file 1: Table S3) hint late to recent gene flow. The Kriging contour maps suggest two paths linking the patterns of diversity of O3a2*-P201, namely from mainland SEA towards Taiwan and from mainland SEA to Indonesia through the Indochinese peninsula (Figure 3). However, these latter results must be viewed with caution given the general low frequency of O3a2*-P201 over the whole region.

Haplogroup O3a2b*-M7 is uncommon in the Fujian Han (less than 2%), in Taiwan (only observed at low frequencies in TwHan and TwPlt, not observed among TwMtA) and the Philippines, but was more frequent in Indonesia (8.54%) (Figure 2 and Additional file 1: Table S2). In this study we observe it also in Indochina (12.5% and 6.7% in Vietnam and Thailand, respectively), as well as in Malaysia (although this latter region is represented by a sample of only 8 Y chromosomes). Age estimates of O3a2b*-M7 diversity for Indonesia (22.18 ± 6.27 Kya) and TwPlt (16.56 ± 4.67) are somewhat older than that for Thailand (12.42 ± 3.45) (Additional file 1: Table S3). However, as already noted, these estimates are based on the assumption that no diversity is introduced in a clade by gene flow between populations. However, the scattered and generally derived location of Indonesian, TwPlt and Filipino haplotypes in the O3a2b*-M7 MJ network is consistent with repeated introductions of these haplotypes by gene flow from the mainland into the islands (Figure 4).

Haplogroup O3a2c*-P164 is frequently observed among TwMtA on the east coast of Taiwan (Amis 35.9%, Puyuma 13%, Taroko 5%), in the Philippines (from 8% to 50%) and in western Indonesia (17.8%, Kalimantan 20%, and Sulawesi 11.8%), while it is rather uncommon in Indochina and among the Han (Figures 2 and 3, and Additional file 1: Table S2). Interestingly, the star-like MJ network of O3a2c*-P164 shows core nodes mostly comprised of Han, Korean/Japanese and Indochinese groups (Figure 3) and radiating sectors mainly comprised of either Korean/Japanese and Tibet, or Indochinese, or delineating TwMtA. This structure supports the dispersal paths put forward in the contour map (Figure 4).

In turn, derived haplogroups O3a2c1*-M134 and O3a2c1a-M133 are rarely seen among TwMtA. They often occur together and are more frequent among Han and TwPlt as well as in Sumatra, Borneo and the Visayas in the Philippines. Age estimates of the diversity of these two haplogroups all point to the early Neolithic period (i.e. < 10,000 Kya), with the notable exception of estimates for the Han groups (Table 2 and Additional file 1: Table S3). Accordingly, the contour maps of O3a2c1*-M134 display a general location of higher frequency and diversity related to Han populations, in mainland SEA (Figure 3).

Finally, haplogroups C, D, F*, H, K*, N, P*, Q and R haplogroups were observed at low frequency in the region, with patchy distributions (Figure 2 and Additional file 1: Table S2).

### Patterns of differentiation of SEA populations

**Figure 5:**
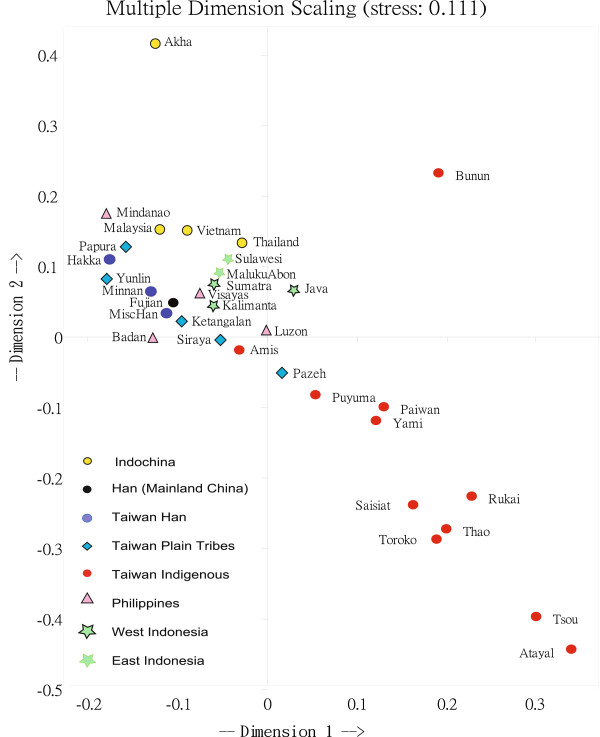
**Multidimensional scaling plot of Reynolds pairwise population genetic distances using high definition Y-SNP genotypic data.** The stress value is 0.111.

The plot of the multidimensional scaling (MDS) analysis of pairwise Reynolds genetic distances obtained from high definition Y-SNP haplogroup frequencies in our dataset is shown in Figure 5. Most population samples are located in the center of the plot, with Austronesian speaking groups from Indonesia and the Philippines surrounded by populations from Vietnam, Thailand and Malaysia (differentiating towards the upper-left part of the plot), most TwMtA differentiating towards the bottom-right, and the Han, both from the mainland and from Taiwan, differentiating towards the upper-left along the x axis. The heavily sinisized TwPlt surround Han populations with Yulin and Papora on the left part of the Han, and the Pazeh and Siraya (from Tainan, Pingtung, and Hwalian) getting close to southern TwMtA and most likely representing less sinisized groups than other TwPlt. A relationship with the frequencies of O clades in these populations (Additional file 1: Figures S1 and S2) is evidenced by the fact that O2 haplogroups prevail in Indochinese populations located in the upper-left quadrant of the MDS plot, whereas O1 haplogroups become more and more frequent in Indonesian, Filipino, south TwMtA and north TwMtA populations, which differentiate towards the bottom of the plot. Bunun, Akha, Atayal and Tsou are clearly four distinct outliers in the plot, due to their particularly low diversity. Indeed, only 3 haplogroups were observed among 56 Bunun chromosomes, of which O1a2-M50 at 61% and O2a1a-M88 at 37.5%, and this translates in a gene diversity level of only 0.49 (Additional file 1: Table S2). Also, only 3 haplogroups were observed among 41 Tsou and 52 Atayal chromosomes, of which O1a1*-P203 at 90% (amongst the highest frequencies observed in ISEA for this haplogroup), with a gene diversity of 0.18 for the two groups. As for the Akha population, its differentiation from all other SEA populations is driven by the unique presence of haplogroup Q-M242 (56%), which was mainly unobserved in our dataset, except for a very low frequency (<1%) in TwHan. The axis of differentiation constituted by TwMtA in the lower-right part of the MDS plot is mostly driven by the northern tribal groups (Taroko, Thao, Saisiyat and Atayal) and underlines their low gene diversity (*h* is 0.10, 0.23, 0.23 and 0.18, respectively) due to the high frequency of haplogroup O1a1*-P203 (Additional file 1: Figure S1 and Additional file 1: Table S2).

In a second MDS analysis, we included earlier published data (Additional file 1: Table S1) to investigate the genetic relationships of populations represented in our dataset to other East Asian groups. The resulting 2-dimensional plot is provided in Additional file 1: Figure S4. The range and variation of Y chromosome diversity present in Taiwan is well exposed in this second MDS, which echoes the first one reported in Figure 5. Taiwanese tribes, starting first with the Amis, then the southern TwMtA tribes and finally the northern TwMtA tribes spread away towards the lower-right part of the plot, from a lower central cluster principally comprising TwPlt, the Philippines, Western Indonesia, some Han from southern China and a few Daic-speaking groups. The latter also form a second axis of differentiation including mainly Daic-speaking groups from China, as well as Indochinese populations that spreads towards the lower right-part of the plot. With respect to Figure 5, the outlier location of the Bunun is again indicative of a high level of genetic drift in this population, consistent with its low level of gene diversity. Interestingly enough, however, the position of the Bunun in the MDS, at midway between the axis of differentiation of TwMtA and that of the Daic-speaking groups from southern China, is explained by the high frequencies of haplogroups O1a2-M50 (67%, the highest frequency seen in Taiwan) and O2a1a-M88 (37%), both of which are commonly found among Daic populations in south China [27].

### Admixture

Contribution from two putative parental groups, namely “ancestral Han” and “ancestral Austronesians”, to a hybrid population, respectively, TwMtA, TwPlt, Filipinos and Indonesians, estimated by using Admix 2.0 and through the STR lineage sharing method (LS) are reported in Table 3. Frequencies of Y-STR lineages for Admix and LS were obtained in the background of their respective Y-SNP haplogroup.

**Table 3:** Gene contribution estimates between populations using variation at 7 Y-chromosome STR loci

**Putative parental groups**	**Admix 2.0**				**Lineage sharing (LS)**			
	**Hybrid populations**				**Hybrid populations**			
	**Philippines (n = 144, hp = 127)**	**TwMtA (n = 350, hp = 156)**	**Indonesia (n = 222, hp = 194)**	**TwPlt (n = 347, hp = 206)**	**Philippines (n = 144, hp = 127)**	**TwMtA (n = 350, hp = 156)**	**Indonesia (n = 222, hp = 194)**	**TwPlt (n = 347, hp = 206)**
Han All (n = 299, hp = 233)	0.38 ± 0.08	0.35 ± 0.03	0.30 ± 0.08	0.62 ± 0.08	0.13 (0.04)	0.08 (0.03)	0.14 (0.06)	0.79 (0.12)
Austronesian pool (n = 716, hp = 458)	0.62 ± 0.08	1.35 ± 0.07	0.70 ± 0.085	0.38 ± 0.08	0.87 (0.26)	0.92 (0.33)	0.86 (0.39)	0.21 (0.03)

Using Admix 2.0 [52,53], the time in years to the admixture event and the mutation rate per year were set to zero. The contribution from the putative parental population is expressed by the estimator of admixture mγ (±standard error). Note that the sum of parental contribution sums to one. The greatest estimate of mγ determines the greater contributor [69]. For lineage sharing [53] the contribution also sums to one. ( ) The observed relative frequency of the contribution to the hybrid population is indicated in brackets. hp = number of 7xSTR haplotypes.

Results between Admix and LS (Table 3) correlate well and suggest that all putatively hybrid groups but the TwPlt received an important contribution from “ancestral Austronesian” speaking populations. In turn, TwPlt Y-SNP haplogroups are predominantly shared with Han populations (Figure 2 and Additional file 1: Table S2) and expectedly the “ancestral Han” contribution estimated with Admix and LS is high (62% and 79% respectively). However, the effective relative frequency of parental Y-STR haplotypes contributed by each parental group, as estimated through the LS method (shown in brackets in Table 3), reached a lower level than anticipated, with only 12% of TwPlt Y-STR variation attributed to the “ancestral Han” gene pool and only 3% to the “ancestral Austronesian”. This indicates that 79% (i.e. 44/ 209) of the remaining Y-STR variation is unique to the TwPlt. Such a large amount of unshared variation could only have been acquired after a long period of settlement in isolation from other groups, much longer than 400 years, date at which Han Chinese (Minnan and Hakka) migrated to Taiwan from southeast China [1]. Actually, on the basis of MJ networks constructed using only the unshared haplotypes showing continuity in the background of their respective haplogroups we tentatively estimated this period to ~3 to 8 Kya.

### Analysis of molecular variance

Analysis of molecular variance (AMOVA) was performed first using Y-SNP haplogroups and then Y-STRs haplotypes (Table 4). The TwMtA group shows the lowest SNP variance within populations (70.3%), consistent with the low gene diversity observed in this group of populations, ranging from 0.10 to 0.7, or averaging to 0.60 ± 0.02 overall (Additional file 1: Table S2). Accordingly, the TwMtA also display the highest SNP variance due to differentiation between tribes (29.7%) thus explaining the scattered location of the Taiwan tribes observed in the MDS plot (Figure 5 and Additional file 1: Figure S4).

**Table 4:** Analysis of Molecular variance (AMOVA) using Y-SNPs haplogroups and 17 Y-STRs for various groupings of populations

**Grouping**		**Variation seen**					
		**Among populations**		**Within populations**			
		**Variance (%)**	**Fst**	**Variance (%)**			
***Y-SNP***	Han (East China)	1.17	0.01	98.83			
	TwMtA (9 tribes)	29.70*	0.30	70.30			
	TwPlt (Siraya, Pazeh)	3.76*	0.04	96.24			
	Ph (Luzon, Mindanao, Visayas)	1.90	0.02	98.10			
	Western IN (Borneo, Sumatra, Java)	5.70*	0.06	94.30			
	Indochina (Thai, Akha, Vietnam)	10.41*	0.10	89.59			
		**Variation seen**					
		**Between groups**		**Among populations within groups**		**Within populations**	**Among all populations considering no grouping**
		**Variance (%)**	**Fct**	**Variance (%)**	**Fsc**	**Variance (%)**	**Fst**
	**Geographic grouping**						
	TwMtA/Ph/Western IN/Han	18.09*	0.05	6.51*	0.14	75.40	0.18*
	TwMtA/Ph/Western IN/Han/Indochina	16.83*	0.17	7.41*	0.09	75.76	0.24*
	**Linguistic grouping**						
	TwMtA/Ph + Western IN/Han	18.55*	0.19	6.97*	0.086	74.48	0.26*
	TwMtA/Ph + Western IN/Han/Indochina	17.19*	0.17	7.21*	0.09	75.59	0.24*
***Y-STR***		**Variation seen**					
		**Among populations**		**Within populations**			
		**Variance (%)**	**Fst**	**Variance (%)**			
	Han (East China)	0.18	<0.01	99.82			
	TwMtA (9 tribes)	31.78*	0.32	68.22			
	TwPlt (Siraya, Pazeh)	3.99*	0.04	96.01			
	Ph (Luzon, Mindanao, Visayas)	−0.84	−0.01	101.00			
	Western IN (Borneo, Sumatra, Java)	2.34*	0.02	97.66			
	Indochina (Thai, Akha, Vietnam)	11.26*	0.11	88.73			
		**Variation seen**					
		**Between groups**		**Among populations within groups**		**Within populations**	**Among all populations considering no grouping**
		**Variance (%)**	**Fct**	**Variance (%)**	**Fsc**	**Variance (%)**	**Fst**
	**Geographic grouping**						
	TwMtA/Ph/Western IN/Han	2.74*	0.03	12.99*	0.13	84.25	0.16*
	TwMtA/Ph/Western IN/Han/Indochina	2.98*	0.03	12.74*	0.13	84.28	0.15*
	**Linguistic grouping**						
	TwMtA/Ph + Western IN/Han	2.53*	0.03	13.29*	0.14	84.16	0.16*
	TwMtA/Ph + Western IN/Han/Indochina	2.79*	0.03	13.01*	0.13	84.20	0.16*

*P < 0.05 AMOVA test for Y-STR was based on 17 STR loci TwMtA: Taiwan mountain tribe Aborigines; TwPlt: Taiwan plain tribes; Ph: Philippines (Luzon, Mindanao, Visayas); Western IN: Western Indonesia (Borneo, Sumatra Java, Sulaweisi).

In contrast to TwMtA, high SNP variation between individuals within populations is found for Western Indonesia (94.30%), the Philippines (98.10%) and TwPlt (96.24%), and the levels of differentiation among populations within groups are correspondingly low, ranging from non-significant for Filipino populations to less that 6% among western Indonesians, even though populations in Western Indonesia and the Philippines are broadly dispersed over many isolated regions of ISEA. On another other hand, the high variance seen within TwPlt populations (96.24%) was expected as they are heavily admixed groups.

When testing genetic differentiation among four separate geographical groups, namely TwMtA, Philippines, Western Indonesia and Han (representing mainland China), or three distinct language family assortments (Formosan, Malayo-Polynesian and Sino-Tibetan), we observe that the variation between groups is large (18% and 18.55% respectively, P < 0.001) and did not show much difference between geographic or linguistic groupings. This pattern remains the same when including Indochina as a fifth geographic region or as a fourth linguistic group.

Very little changes from the Y-SNP results were observed when using Y-STRs to perform AMOVA computations on the individual groups of populations, with the variance due to differences between individuals within populations being extremely high for TwPlt, Philippines and Western Indonesia (above 96%) and much lower for TwMtA (68%). However, with Y-STRs we observe that the component of the variance due to differences between groups of populations, although significant, is always lower than that due to differences among populations within these groups, thus indicating that, contrarily to Y-SNPs, Y-STRs fail to detect a population structure associated with geography or linguistics.

## Discussion

Our data, obtained through the genotyping of 81 high Y-SNP definition markers to determine the fine distribution of Y-chromosome haplogroups O in ISEA, revealed a high level of population structure in the region including Taiwan, the Philippines and Indonesia, mainly defined by variable distributions of haplogroups belonging to the O clade [9,27,70]. We also genotyped 17 Y-specific STR markers, in order to gain insights into the distribution of the Y-chromosome variation in this region of the world. Since only one population from the mainland east coast of China (Fujian Han) was analyzed, data from other populations of SEA were obtained from the literature (Additional file 1: Table S1).

The O clade, to which belong 95% of Y-chromosomes in Taiwan, reflects an ancestral relationship to the early modern human settlement in East Asia [28-30] with haplogroup, O1 being mostly seen in Southeast China, Taiwan and ISEA, haplogroup O2a predominantly confined to southeast China and Indochina, and haplogroup O3 broadly present in mainland China.

### Haplogroup diversity in Taiwan and its relation with the Philippines and Indonesia

The Y-SNP gene diversity among TwMtA was found generally low (from 0.1 to 0.7, Additional file 1: Table S2). Except for the presence of haplogroups O2a1a-M88 predominantly seen in Bunun (37.5%) and O3a2c-P164 in Puyuma and Amis, the low diversity found in TwMtA is principally associated with the molecular variation of haplogroups within O1 (Figure 2 and Additional file 1: Table S2 and Additional file 1: Figure S1). In sharp contrast with the TwMtA, non-aboriginal groups in Taiwan, namely Minnan, Hakka and the general mixed Han Taiwanese (MiscHan), as well as the TwPlt groups all present high levels of gene diversity, and a total of 26 distinct branches of the O clade (including the O1, O2 and O3 clades) were observed in these groups at variable frequencies. These results suggest substantial admixture among plain tribe groups in Taiwan, not among mountain tribes. Fast genetic drift in TwMtA, due to small population size and isolation is a likely explanation for these observations, and a founder event linked to the initial settlement of the island is also plausible. On another hand, larger population sizes in TwPlt, possibly related to gene flow and admixture events, would have resulted in the higher levels of diversity in TwPlt observed nowadays. Alternatively, the possibility exists that our village-focused sampling method for TwMtA might have biased our results towards underestimation of the actual diversity present in these groups. Similarly, we cannot exclude that very recent migration to main urban centers from where most of our sampling comes from must have contributed toward the greater diversity observed in the Philippines and western Indonesia. However, to our credit, the scattered location of TwMtA groups observed in the MDS plot (Figure 5 and Additional file 1: Figure 3), which strongly suggests fast genetic drift, matches similar patterns reported elsewhere for Taiwan and also for Melanesia [67,71,72]. Furthermore, a similar scattered pattern was also observed with polymorphisms in HLA loci [73], see Figure 3 in it), as well as with a classical marker [74], thus further supporting the idea that the genetic history of TwMtA was characterized by drift occurring in small isolated groups.

In a recent study of the diversity of Taiwan mtDNA complete genomes, Ko et al. [75] observed a decreasing pattern of diversity in TwMtA populations from north to south, although the significance of the decrease was not reported. A similar tendency for diversity to decrease towards southern Taiwan was also found for the classical GM genetic polymorphism but did not reach statistical significance [74]. Interestingly, for the non-recombining Y chromosome, we find here an opposite pattern in that gene diversity increases from north to south in a significant fashion (Additional file 1: Figure S3, first plot, *P* = 0.0142). This increase is concomitant with a tendency for decreasing frequency of haplogroup O1a1* − P203 and increasing Y-STR diversity in this haplogroup, but both these patterns are statistically not significant (Additional file 1: Figure S3). Although the contrasting results between mtDNA and autosomal markers, on one hand, and the Y chromosome on the other are for the time being inconclusive, they nevertheless could hint to a differentiated demographic history of men and women in TwMtA.

Haplogroup O1a1*-P203 was seen at high frequencies, ranging from 40% to 60% in all southern and eastern TwMtA (Paiwan, Puyuma, Paiwan, Rukai, Amis and Yami), and was above 87% in all northern and central mountain tribes (Atayal, Taroko, Saisiyat, Thao and Tsou). Conversely, O1a1*-P203 rarely exceeds 20% in the Philippines and Indonesia (Figures 2 and 3, and Additional file 1: Table S2 and Additional file 1: Figure S2), but it has been reported as common in south and east China where it most likely originated [42]. We note also that the para-haplogroup O1a1a-M101 was not observed in our dataset covering ISEA, and thus extend in this way knowledge from previous reports indicating that it must be rare in most regions of continental East Asia [30,42,62,65].

With respect to O1a1*-P203, it is possible that this haplogroup reached Indonesia through gene flow from the mainland, via the Indochinese peninsula. We note however that O1a1*-P203 is uncommon in Indochina. Moreover, the presence of O1a1*-P203 among the Korean [76], the Han from Fujian and TwHan (Additional file 1: Table S2) rather argue for a common origin in an ancient dispersal from the mainland (Figure 3).

A second, major O-clade haplogroup observed in Taiwan is O1a2-M50, where it is frequent in several Aborigine populations, especially so among southern TwMtA, but rather rare in the Han populations of the island. As this haplogroup has been reported as frequent (from 3% to 25%) among some Daic-speaking groups from southern China and Hainan, it was suggested that its expansion throughout ISEA followed an OOT model of migration reaching sequentially, Taiwan, the Philippines and Indonesia [26,65]. Our results support this hypothesis by demonstrating a decrease in frequency and molecular variation of O1a2-M50 from Taiwan to the Philippines and Indonesia (Figure 4, Table 4 and Additional file 1: Table S2). The pattern of STR variation is compatible with a late Paleolithic origin of this haplogroup in SEA with migration and expansion in Taiwan (~17 Kya), followed by pre-Holocene passages to the Philippines (~12 Kya) and Indonesia (~7 Kya) (Figure 3).

A remarkable result emerging from our dataset is the high frequency of the O2 clade, more specifically of haplogroup O2a1a-M88, found in the Bunun (37.5%, Additional file 1: Table S2). Except for the presence of O2a1a-M88, as well as O2a1*-M95 in the Yami, this is the only detected occurrence of the O2-clade in a TwMtA group. Of course, the absence of O2 in a sample does not exclude its presence in the population but it does suggest that it is likely less frequent. We observe that the Bunun share their most common Y-STR haplotype of O2a1a-M88 with the Daic- speaking groups and with the Indochinese and Indonesian populations, while they share none of the haplotypes found among Solomon islanders, TwPlt and Han (Figure 4). Actually, the Bunun STR haplotypes do not belong to the same branch as the Solomon Islands or Madagascar [7,66,67]. To our opinion, these results, together with the observed scarceness of the O2 clade among TwMtA and the Philippines, preclude the TwMtA as a plausible contender for the paternal origin of O2 haplogroups in Madagascar and the Solomon Islands, although the possibility that the O2 clade was lost by drift in most Austronesians from Taiwan and in the Philippines remains.

Haplogroup O3 constitutes the molecularly most diversified clade observed in continental East Asia [7,8,26-31,40,68]. The introduction of new Y-SNP markers for better assignment of O3 subtypes allowed us to demonstrate several characteristics that were not seen before [8,43,44]. Haplogroup O3a1c*-IMS-JST002611 has been reported in Japan, Korea, Tibet, south China and Indochina [62,65,76,77]. Its high prevalence observed in this study in Fujian Han and Taiwan Minnan and Hakka (14% to 26%), but very low occurrence among TwMtA (Yami, 3%) and in the Philippines (0.8%), likely represents a signature of Han-mediated gene flow. Indeed, the lack of a clear clinal pattern of frequency variation of this haplogroup between regions, the low frequency but high diversity observed in Indonesia (Figure 2 and Table 2) and the rather faint star-like shape of the MJ network showing numerous long branches and low frequency nodes (Figure 4) all suggest a recent spread of O3a1c* from mainland SEA. Thus the O3a1c* high frequency observed for the Ivatan (25%) of the Batan archipelago, north of the Philippines, associated to a low age estimate (0.9 Kya, Table 2 and Additional file 1: Table S3), is consistent with a recent introduction of this haplogroup by gene flow with TwHan or TwPlt. The presence of Han lineages at most nodes of the star-like MJ network supports this hypothesis (Figure 4).

Both the frequency distribution of haplogroup O3a2b*-M7 (which is present at low frequencies in Taiwan, among the Han and TwPlt) and its MJ network are consistent with repeated introductions of this haplogroup by gene flow from mainland Southeast Asia into the islands (Figure 4). As already stated, we observe this haplogroup at frequencies of 7% or higher in mainland SEA. Previous studies also reported the presence of this haplogroup in SEA, in Yunnan, Fujian, and among the She and Yao ethnic groups [30,40]. On another hand, haplogroup O3a2c*-P164, whose origins also likely trace back to mainland SEA, is quite frequent in Taiwan (especially so among the Amis), whereas the derived O3a2c1*-M134 and O3a2c1a-M133 are rather rare among TwMtA. In our dataset we observed that they often occurred together (Additional file 1: Table S2), and given the recent dates estimated for their STR diversification (~8 to 6 ± 4 Kya ago) (Figure 3, Table 2 and Additional file 1: Table S2), it is likely that their spread southwards to Vietnam, Laos and Thailand, as well as to Taiwan, the Philippines and Indonesia, concomitant with a northward spread to Japan [26,77], occurred during Neolithic times.

### The pincer model and the four stages of migration

It has been proposed that, after the initial colonization approximately 50 Kya of SEA and Indochina by modern humans bringing along haplogroups C, M and S, the origin and spread of most haplogroups seen today in east Asia, Taiwan and ISEA could be retraced according to four stages (A to D) of a paternal demographic model [26]. Stages B, C and D correspond to gene dispersals taking their origin in mainland Southeast Asia or Indochina and whose directions of flow form a pincer model, with a northern branch spreading through Taiwan and a southern branch through Indonesia. In this pincer model, the Philippines appear as a confluent region of genes acquired separately from Taiwan or Indonesia and more recently from the Asian mainland. The patterns of genetic diversity observed in this study are consistent with this scenario in that several haplogroups displaying a cline with lower Y-STR diversity over Western Indonesia (i.e. O1a*-M119, O1a1*-P203 and O1a2-M50) or lower diversity over Taiwan (O2a1*-M95) show diversity higher than expected in the Philippines, as attested by the corresponding age estimates (Table 2 and Additional file 1: Table S3).The northern branch postulated by the pincer model would have first reached Taiwan, thus introducing in the island haplogroups O1a*-M119, O1a1*-P203 and O1a2-M50. Further, these haplogroups also display a decreasing cline in frequency from Taiwan towards the Philippines and Western Indonesia (Figure 3).

The MJ networks associated with the O1 clade are the only ones in which Y-STR haplotypes of TwMtA are observed among the f0 founder nodes (Figure 4). Along with the very low frequency of non-O1 clades among TwMtA, these results suggest that haplogroups O1a1 and O1a2 represent the earliest traces of the Austronesian-agriculturist dispersal to Taiwan. Furthermore, TwMtA haplotypes of other haplogroups (O2-P31/O2a-Pk4/O2a1-M95; O2a1a-M88; O3a2b-M7 and O3a2c-P164) were rarely seen among the f1 founder nodes, consistent with the hypothesis that later direct east–west sea passages occurred between mainland East Asia and Taiwan. Thus, the Y-SNP haplogroups frequency distributions and their STR diversity observed today in TwMtA populations give support to the northern branch of the pincer model (the Taiwanese branch). This Austronesian-agriculturist dispersal most likely expanded principally within the boundaries of present-day Taiwan and the Philippines before reaching Western Indonesia which was already populated by indigenous hunter–gatherers, possibly of Asiatic origin [64].

Populations that first went south from Southeast Asia along or from the Indochinese peninsula, Malaysia, western Indonesia (Sumatra, Java, and Borneo) and the Philippines represent the southern branch of the pincer model. This dispersal would include haplogroups O1a1*-P203, O2a1-M95/M88, O3a*-M324, O3a1a-M121, O3a1c*-IMS-JST002611, O3a2*-P201 , O3a2a-M159, O3a2b*-M7, O3a2c*-P164 and O3a2c1a-M133 [14,26,27,67,78]. For most of these haplogroups, it is currently held that they first expanded and diversified within the boundaries of present-day southeast China, Indochina and Indonesia, and they are considered as involved in a Paleolithic contribution from mainland Asia by Karafet et al. [26].

The tips of the two branches of the pincer model may have, at times, reached and crossed over in the Philippines. Such bidirectional dispersals have been previously proposed on the basis of specific patterns of mtDNA diversity, namely the northward spread of mtDNA haplogroup E from the Philippines to Taiwan through gene flow [17] and the southward frequency gradient of mtDNA haplogroup B4a1a [18,19,79]. This scenario is consistent with, although not statistically reinforced by the unexpected increase in diversity of haplogroups O1a1-P203 and O3a2c-M164 observed in the Philippines (Figure 3, Table 2 and Additional file 1: Table S3).

The pincer model described here applies mostly to western ISEA. The two branches rejoin further east through ISEA where significant eastward decreasing frequency clines of most haplogroups of the O clade have been observed, although with resurgence of some derived haplogroups of the O3 clade in the Bismark archipelago and Polynesia [80,81]. Most likely, part of this scenario may be ascribed to the “out of Taiwan” model [26].

Our results are in agreement with the multidirectional gene flow out of SEA described by Li et al. for haplogroup “O1a*”, which was further resolved here as O1a1*-P203 [26,27]. Although the genetic relationships of the Philippines with Taiwan and Western Indonesia are well supported in this study on the basis of Y-SNP variation, the fast mutation rate of Y-STRs, together with likely long periods of isolation of the groups, contributed to more distinct structuring between regions and low present-day haplotype sharing (Figure 4 and Tables 2 and 4) [32-34]. Indeed, the significant genetic differentiation observed between regions of the mainland and western insular SEA evidenced by AMOVA analyses would not be expected if substantial gene flow between these regions would have been maintained after the initial dispersals held by the pincer model (Table 4). Furthermore, the rare instances of sharing of Y-STR haplotypes observed between distant regions, bypassing regions in between (i.e. the occurrence of nodes shared uniquely between Indonesia and TwMtA in the O1a1-P203 MJ network, Figure 4), may be the product of later admixture through significant maritime trade activities [82,83], rather than admixture resulting from land driven migration of peoples where gene continuity in the MJ networks should have been evidenced (Figure 4).

### Correspondences with the four stages of dispersal model

Consistent with the first stage (A) of Karafet et al. [26], we found that ancient traces of the initial settlement of modern humans in East Asia ~50 Kya ago were retained in the Philippines, through the occurrence of haplogroups F* and K* in this archipelago. While possibly coming from SEA, the higher frequency of haplogroup K in the Philippines than in Indochina and Southeast Asia [11,36] suggests that the presence of two individuals in Taiwan bearing this haplogroup could result from gene flow between the Philippines and Taiwan [18].

More generally, the presence of haplogroups C, K* or F* in Western Indonesia may be associated with the Paleolithic colonization of ISEA, whereas that of haplogroups R and H is likely linked to more recent migrations from Arabs, Indians (or Eurasians) and need to be determined further. Lastly, haplogroups D, N, P* and Q-M242 may be associated with isolated migrations from central Asia [8,14,50,84]. Since the Akha made their way from China into South East Asia during the early 1900s the prominence of Q-M242 in the Akha (56%) is intriguing and more specific determination of this haplogroup should reveal if a central Asian origin and spread into SEA is supported or whether Q* was introduced from the south, coming from the Indian subcontinent [84,85].

We confirm stage B of Karafet et al. [26] coalescence results indicating that dispersal within SEA may have initiated in late the Paleolithic since the age estimates of the diversity of haplogroups O1a*, O1a1*, O2a1* and O3* inferred here for the whole region are of 18 Kya to 14 Kya (Figure 3).

Stage C describes later migrations/gene flow, between approximately 8 to 6 Kya ago, corresponding to the early Neolithic Austronesian expansion and that brought not only O1a1*-P203, O1a2-M50/M110 and O3a2*-P208 [26] but also O2a1*-M95 (nowadays observed in the TwPlt), O2a1a-M88 (observed in the Bunun), and O3a2c*-P164 to Taiwan, the Philippines and near Oceania, the latter most likely in association with the Out of Taiwan dispersal 4 Kya ago (Figure 3).

In addition to O3a2b*-M7 in stage D of Karafet et al. [26], we propose to consider also O3a*-M122 as a late introduction in Taiwan from the mainland, as well as haplogroups O3a1c-2611 and O3a2c1-M134 which could have reached Taiwan and Western Indonesia separately, through the northern and southern dispersal branches of the pincer model, respectively.

### Genetic relationships with Madagascar and the Solomon islands

Madagascar and the Solomon islands may share a common paternal molecular past. While haplogroup O1a2-M50 is commonly seen in Taiwan and moderately distributed throughout western ISEA, O2a1-M95 is rare all over the Philippines [7], common in Western Indonesia [7] and even more so in Indochina. Interestingly, haplogroups O1a2-M50 and O2a1-M95/M88 were seldom observed together in the same population in our dataset or in published data [7]. Their co-occurrence in four well separated groups, Madagascar, the Solomon Islands, Bunun and Western Indonesia is intriguing and most likely implies a complex relationship between a series of male migration events from Western Indonesia [66,68,81,86]. We note that the core node (f0) of the O2a1-M95/M88 MJ network (Figure 4) comprises haplotypes presently shared between Indochinese, western Indonesians, Han, Daic groups on the mainland (and Hainan) [27,36] and a few TwPlt, but not the Bunun whose founder haplotype belongs to an f1 isolated node containing uniquely O2a1a-M88 chromosomes. It is possible that the missing O2a1a-M88 among all TwMtA in Taiwan except for the Bunun is due to an isolated migration event from mainland Southeast Asia that did not affect already isolated TwMtA tribes. By contrast, the presence of O2 haplogroups in the Yami is most likely due to later gene flow between Taiwan and Tao Island (Orchid island) [25].

We also found that all nodes leading to the Solomon Islands in the O2a1-M95/M88 MJ network include Y-STR haplotypes also observed in Madagascar (Figure 4). In other words, the MJ networks of O1a2-M50 and O2a1-M95/M88 provide clear evidence of a genetic footprint of common ancestry between Malagasy and Solomon islanders. It is a generally accepted view that O1a2-M50 traces its origin back to Taiwan [63] which leads to the assumption that O2a1-M95/M88 followed the same route. But considering the scarcity of O2a1-M95/M88 in the Philippines, it is likely that haplogroups O1a2-M50 and O2a1-M95/M88 reached Indonesia through separate dispersals, O1a2-M50 through the northern branch of the pincer model and O2a1-M95/M88 through its southern branch, and that these branches coupled in Western Indonesia, possibly in Borneo or Sumatra. Later migrations drove this haplogroup, as well as O3a2c-P164, eastwards to the Solomon Islands, and westwards to Madagascar. These migrations could have also taken along mtDNA haplogroups F3b and M7c3c [14,18,19,66], and may have happened at the time when the Malagasy motif of mtDNA haplogroup B4a1a1a (with mutations at np. 1473 and 3423) appeared [86].

### The Taiwan plain tribes

Lastly, we found that the Taiwan plain tribes share more Y-STR haplotypes with Taiwanese Han (12%) than with TwMtA (3%) (Table 3, LS relative frequency). On the other hand, 77% of Y-STR haplotypes in TwPlt were not shared with any other group. Dating estimates were obtained from these unshared haplotypes in the background of their corresponding haplogroups (O1a1-P203, O1a2-M50 and O3a2c-P164, whose frequencies are of 16%, 6% and 18%, respectively) using MJ sub-network clusters that show continuity between lineages. Values ranging from 2,616 ± 1.806 Kya to 6.458 ± 2.951 Kya (data not shown) were obtained. Although the standard deviation associated with these assignments is large, these results raise the possibility that, rather than having evolved in the TwPlt as a result of long isolation, a significant amount of unique Y-STR diversity may have been acquired from different waves of settlers than those whose descendants are observed today in urban centers.

## Conclusion

Our fine-scale study of Y-chromosome polymorphisms in Southeast Asian populations supports the view that the male genetic diversity of present-day populations living in Taiwan, the Philippines and Western Indonesia was formed through a complex pattern of settlement and dispersals, here coined the pincer model, that perfectly matches expectations derived from previously described models [26]. This pincer model includes two dispersal routes to ISEA, namely a northern route, from the mainland through Taiwan and a southern route through western Indonesia. Age estimates of the diversity of the major Y-chromosome haplogroups observed in SEA all fall approximately within the last 20 Kya. This puts an upper bound to the starting time of dispersals in the pincer model that is in agreement with previous studies that concluded to a settlement history of ISEA starting in the late upper Paleolithic and continuing during the early Neolithic, notably with the expansion of Austronesian peoples [14,18,26,27,87]. It is during this last period that gene flow between specific islands likely intensified, notably in the Philippines, where high genetic diversity of several Y-SNP haplogroups seems to be due to independent gene flow with either Taiwan or Indonesia. A further likely contribution to the high Y-chromosome diversity in the Philippines is gene flow due to sailing traders from the southeast coast of China or Eastern Indochina (Vietnam) [82]. However, genetic drift due to the late upper Paleolithic to early Neolithic isolation of populations settling in the islands may have substantially contributed to the significant differentiation of male Y-SNP and Y-STR pools between SEA regions. On another hand, it was found that Madagascar and the Solomon Islands might share part of their paternal ancestry in Western Indonesia (or the southern Indochinese peninsula), in a population having acquired haplogroups O1a2-M50 from Taiwan and O2a1-M95/M88 from Indochina. Lastly, although the paternal genetic pool of the Taiwan plain tribes closely resembles that of Han populations from Taiwan and Fujian, the high diversity of non-shared haplotypes found in the plain tribes suggests a settlement in Taiwan that pre-dates the arrival of Han in the island by some 2 to 6 Kya.

In the future, complete human genome data analysis and more pertinent and larger sampling of populations should help in obtaining a better assessment of drift, admixture and the timing of migrations events between continental East Asia, Taiwan, and other regions associated with the wide dispersal of the Austronesian societies.

## Abbreviations

TwPlt: Taiwan plain TRIBES; MiscHan: A Taiwan Han group composed of miscellaneous Minnan individuals who were uncertain about the origin of at least one of their parents; TwHan: Taiwan Han; Minnan: Hakka and MiscHan; TwMtA: Taiwan Mountain tribe Aborigines; Kya: Thousand years ago; SEA: Southeast Asia; ISEA: Island Southeast Asia; MSEA: Mainland Southeast Asia/Indochina.

## Competing interests

The authors declare that they have no competing interests.

## Authors’ contributions

JAT and ML conceived and designed the experiments. JCY, CLL and JHL performed the experiments. JAT analyzed the data, and ESP participated in the analyses. ML contributed reagents/materials/analysis tools. JAT and ESP wrote the paper. All authors read and approved the final manuscript.

## Supplementary Material

Additional file 1: Figure S1 Frequency distributions of O clades (O1, O2 and O3) in samples from Taiwan and two neighboring populations (Fujian Han, and Ivatan from Batan). **Figure S2.** Frequency distributions of O clades (O1, O2 and O3) in pooled samples from Taiwan compared to the Philippines, Indonesia and mainland Southeast Asia. **Figure S3.** Variation of diversity measures according to latitude in TwMtA. The three plots display the estimated values and linear regression of, respectively, gene diversity (Additional file 1: Table S2), frequency of haplogroup O1a1*-P203 (Additional file 1: Table S2), and STR diversity of haplogroup O1a1*-P203 (measured by the rho statistic, Additional file 1: Table S3), on latitude. Pearson’s linear correlation coefficient and its statistical significance are given in the respective three captions. **Figure S4.** MDS plot (stress 0.203) using our data and literature data from Additional file 1: Table S1 (over 6000 chromosomes). Haplogroup frequencies were adjusted to 20 basal haplogroups (low definition SNP). See Additional file 1: Table S1 for correspondence of numbers and populations. **Table S1.** Asian population data from this study and from previously published studies. Data used for MDS analysis shown in Additional file 1: Figure S3. **Table S2.** Frequency distributions and gene diversity of Y-SNP haplogroups in populations from Taiwan, Island Southeast Asia and Indochina. **Table S3.** Age (in 1000 years) and Standard Error (SE) of Y-STR variation within Haplogroups (7 STRs). **Table S4.** Y-SNP and Y-STR raw data. Click here for file

## References

[B1] Chiung WVT Language attitudes toward written Taiwanese J Multiling Multicult Devel 2001 22 502 523

[B2] Murdock GP Genetic classification of the austronesian languages: a key to oceanic culture history Ethnology 1964 3 117 126

[B3] Blust R The prehistory of the Austronesian-speaking peoples: the view from language J World Prehistory 1995 9 453 510

[B4] Bellwood P Prehistory of the Indo-Malaysian Archipelago 1997 Honolulu: HI: University of Hawaii Press

[B5] Diamond J Bellwood P Farmers and their languages: the first expansions Science 2003 300 597 603 12714734 10.1126/science.1078208

[B6] Underhill PA Kivisild T Use of Y chromosome and mitochondrial DNA. Population structure in tracing human migrations Annu Rev Genet 2007 41 539 564 18076332 10.1146/annurev.genet.41.110306.130407

[B7] Delfin F Salvador JM Calacal GC Perdigon HB Tabbada KA Villamor LP Halos SC Gunnarsdottir E Myles S Hughes DA Xu S Jin L Lao O Kayser M Hurles ME Stoneking M De Ungria MC The Y-chromosome landscape of the Philippines: extensive heterogeneity and varying genetic affinities of Negrito and non-Negrito groups Eur J Hum Genet 2010 19 224 230 20877414 10.1038/ejhg.2010.162 PMC3025791

[B8] Karafet TM Mendez FL Meilerman MB Underhill PA Zegura SL Hammer MF New binary polymorphisms reshape and increase resolution of the human Y chromosomal haplogroup tree Genome Res 2008 18 830 838 18385274 10.1101/gr.7172008 PMC2336805

[B9] Su B Jin L Underhill P Martinson J Saha N McGarvey ST Shriver MD Chu J Oefner P Chakraborty R Deka R Polynesian origins: insights from the Y chromosome Proc Natl Acad Sci U S A 2000 97 8225 8228 10899994 10.1073/pnas.97.15.8225 PMC26928

[B10] Friedlaender JS Friedlaender FR Reed FA Kidd KK Kidd JR Chambers GK Lea RA Loo JH Koki G Hodgson JA Merriwether DA Weber JL The genetic structure of Pacific Islanders PLoS Genet 2008 4 1 e19 18208337 10.1371/journal.pgen.0040019 PMC2211537

[B11] Cai X Qin Z Wen B Xu S Wang Y Lu Y Wei L Wang C Li S Huang X Jin L Li H Human migration through bottlenecks from Southeast Asia into East Asia during last glacial maximum revealed by Y chromosomes PLoS One 2011 6 e24282 21904623 10.1371/journal.pone.0024282 PMC3164178

[B12] Kim SH Kim KC Shin DJ Jin HJ Kwak KD Han MS Song JM Kim W High frequencies of Y-chromosome haplogroup O2b-SRY465 lineages in Korea: a genetic perspective on the peopling of Korea Investig Genet 2011 2 10 10.1186/2041-2223-2-10 PMC3087676 21463511

[B13] Abbott WG Winship IM Gane EJ Finau SA Munn SR Tukuitonga CE Genetic diversity and linkage disequilibrium in the Polynesian population of Niue Island Hum Biol 2006 78 131 145 17036922 10.1353/hub.2006.0031

[B14] Tumonggor MK Karafet TM Hallmark B Lansing JS Sudoyo H Hammer MF Cox MP The Indonesian archipelago: an ancient genetic highway linking Asia and the Pacific J Hum Genet 2013 58 165 173 23344321 10.1038/jhg.2012.154

[B15] Melton T Clifford S Martinson J Batzer M Stoneking M Genetic evidence for the proto-Austronesian homeland in Asia: mtDNA and nuclear DNA variation in Taiwanese aboriginal tribes Am J Hum Genet 1998 63 1807 1823 9837834 10.1086/302131 PMC1377653

[B16] Hill C Soares P Mormina M Macaulay V Meehan W Blackburn J Clarke D Raja JM Ismail P Bulbeck D Oppenheimer S Richards M Phylogeography and ethnogenesis of aboriginal southeast asians Mol Biol Evol 2006 23 2480 2491 16982817 10.1093/molbev/msl124

[B17] Soares P Trejaut JA Loo J-H Hill C Mormina M Lee CL Chen YM Hudjashov G Forster P Macaulay V Bulbeck D Oppenheimer S Lin M Richards MB Climate change and postglacial human dispersals in southeast Asia Mol Biol Evol 2008 25 1209 1218 18359946 10.1093/molbev/msn068

[B18] Tabbada KA Trejaut J Loo JH Chen YM Lin M Mirazon-Lahr M Kivisild T De Ungria MC Philippine mitochondrial DNA diversity: a populated viaduct between Taiwan and Indonesia? Mol Biol Evol 2010 27 21 31 19755666 10.1093/molbev/msp215

[B19] Trejaut JA Kivisild T Loo JH Lee CL He CL Hsu CJ Li ZY Lin M Traces of archaic mitochondrial lineages persist in Austronesian speaking Formosan populations PLoS Biol 2005 3 e247 15984912 10.1371/journal.pbio.0030247 PMC1166350

[B20] Sagart L Sino-Tibetan-Austronesian: An Updated and Improved Argument 2004 London: RoutledgeCurzon 161 176

[B21] Richards M Oppenheimer S Sykes B mtDNA suggests Polynesian origins in eastern Indonesia Am J Hum Genet 1998 63 1234 1236 9758601 10.1086/302043 PMC1377476

[B22] Cox MP Indonesian mitochondrial DNA and its opposition to a Pleistocene Era origin of proto-Polynesians in Island Southeast Asia Hum Biol 2005 77 no. 2 179 188 16201135 10.1353/hub.2005.0037

[B23] Oppenheimer S Richards M Fast trains, slow boats, and the ancestry of the Polynesian islanders Sci Prog 2001 84 157 181 11732155 10.3184/003685001783238989 PMC10361203

[B24] Bellwood P Dizon E The Batanes archaeological project and the ‘Out of Taiwan’ hypothesis for Austronesian dispersal J Austronesian Studies (Taitung, Taiwan) 2005 1 1 32

[B25] Loo JH Trejaut JA Yen JC Chen ZS Lee CL Lin M Genetic affinities between the Yami tribe people of Orchid Island and the Philippine Islanders of the Batanes archipelago BMC Genet 2011 12 21 21281460 10.1186/1471-2156-12-21 PMC3044674

[B26] Karafet TM Hallmark B Cox MP Sudoyo H Downey S Lansing JS Hammer MF Major east–west division underlies Y chromosome stratification across Indonesia Mol Biol Evol 2010 27 1833 1844 20207712 10.1093/molbev/msq063

[B27] Li H Wen B Chen SJ Su B Pramoonjago P Liu Y Pan S Qin Z Liu W Cheng X Yang N Li X Tran D Lu D Hsu MT Deka R Marzuki S Tan CC Jin L Paternal genetic affinity between Western Austronesians and Daic populations BMC Evol Biol 2008 8 146 18482451 10.1186/1471-2148-8-146 PMC2408594

[B28] Jin L Su B Natives or immigrants: modern human origin in East Asia Nat Rev Genet 2000 1 126 133 11253652 10.1038/35038565

[B29] Shi H Dong Y Wen B C-J X i Chakraborty R Jin L Su B Y -chromosome evidence of Southern origin of the East Asian–specific H aplogroup O3 -M 1 2 2 Am J Hum Genet 2005 77 408 419 16080116 10.1086/444436 PMC1226206

[B30] Xue Y Zerjal T Bao W Zhu S Shu Q Xu J Du R Fu S Li P Hurles ME Yang H Tyler-Smith C Male demography in East Asia: a north–south contrast in human population expansion times Genetics 2006 172 4 2431 2439 16489223 10.1534/genetics.105.054270 PMC1456369

[B31] Zhang F Su B Zhang Y-P Jin L Genetic studies of human diversity in East Asia Philos Trans R Soc Lond B Biol Sci 2007 362 1482 987 995 17317646 10.1098/rstb.2007.2028 PMC2435565

[B32] Hwa HL Tseng LH Ko TM Chang YY Yin HY Su YN Lee JC Seventeen Y-chromosomal short tandem repeat haplotypes in seven groups of population living in Taiwan Int J Legal Med 2010 124 295 300 20179958 10.1007/s00414-010-0425-9

[B33] Wu F-C Chen M-Y Chao C-H Pu C-E Study on the genetic polymorphisms of Y chromosomal DNA short tandem repeat loci applied to analyzing the relative affinities among ethnic groups in Taiwan Forensic Sci Int Genet Suppl Ser 2013 4 e69 e70

[B34] Tsai LC Yuen TY Hsieh HM Lin M Tzeng CH Huang NE Linacre A Lee JC Haplotype frequencies of nine Y-chromosome STR loci in the Taiwanese Han population Int J Legal Med 2002 116 179 183 12111324 10.1007/s004140100236

[B35] Avarez I Royo LJ Fernandez I Gutierrez JP Gomez E Goyache F Genetic relationships and admixture among sheep breeds from Northern Spain assessed using microsatellites J Anim Sci 2004 82 2246 2252 15318720 10.2527/2004.8282246x

[B36] Lewis MP Ethnologue: Languages of the World, vol. Sixteenth Edition 2009 Dallas, Texas USA: SIL International Publications 7500 West Camp Wisdom Road. Online version: http://www.ethnologue.com/; 2009000000

[B37] Li D Li H Ou C Lu Y Sun Y Yang B Qin Z Zhou Z Li S Jin L Paternal genetic structure of Hainan aborigines isolated at the entrance to East Asia PLoS One 2008 3 e2168 18478090 10.1371/journal.pone.0002168 PMC2374892

[B38] Tan S Yang M Yu H Dong Y Shou W Zou J Tang W Guo Y Xiao C Y-chromosome polymorphisms define the origin of the Mang, an isolated population in China Ann Hum Biol 2007 34 573 581 17786593 10.1080/03014460701492237

[B39] Brucato N Mazieres S Guitard E Giscard PH Bois E Larrouy G Dugoujon JM The Hmong Diaspora: preserved South-East Asian genetic ancestry in French Guianese Asians C R Biol 2012 335 698 707 23199638 10.1016/j.crvi.2012.10.003

[B40] Karafet TM Lansing JS Redd AJ Reznikova S Watkins JC Surata SP Arthawiguna WA Mayer L Bamshad M Jorde LB Hammer MF Balinese Y-chromosome perspective on the peopling of Indonesia: genetic contributions from pre-neolithic hunter-gatherers, Austronesian farmers, and Indian traders Hum Biol 2005 77 93 114 16114819 10.1353/hub.2005.0030

[B41] Wen B Li H Lu D Song X Zhang F He Y Li F Gao Y Mao X Zhang L Qian J Tan J Jin J Huang W Deka R Su B Chakraborty R Jin L Genetic evidence supports demic diffusion of Han culture Nature 2004 431 302 305 15372031 10.1038/nature02878

[B42] Dulik MC Osipova LP Schurr TG Y-chromosome variation in Altaian Kazakhs reveals a common paternal gene pool for Kazakhs and the influence of Mongolian expansions PLoS One 2011 6 e17548 21412412 10.1371/journal.pone.0017548 PMC3055870

[B43] Yan S Wang C-C Li H Li S-L Jin L Genographic Consortion An updated tree of Y-chromosome Haplogroup O and revised phylogenetic positions of mutations P164 and PK4 Eur J Hum Genet 2011 19 9 1013 1015 21505448 10.1038/ejhg.2011.64 PMC3179364

[B44] YCC Y Chromosome Consortium, A nomenclature system for the tree of human Y-chromosomal binary haplogroups Genome Res 2002 12 339 348 11827954 10.1101/gr.217602 PMC155271

[B45] ISOGG Y-DNA Haplogroup Tree. International Society of Genetic Genealogy 2009 Version: 4.06, Date: 10 February 2009; Available: http://www.isogg.org/tree/

[B46] Underhill PA Shen P Lin AA Jin L Passarino G Yang WH Kauffman E Bonne-Tamir B Bertranpetit J Francalacci P Ibrahim M Jenkins T Kidd JR Mehdi SQ Seielstad MT Wells RS Piazza A Davis RW Feldman MW Cavalli-Sforza LL Oefner PJ Y chromosome sequence variation and the history of human populations Nat Genet 2000 26 358 361 11062480 10.1038/81685

[B47] Qamar R Ayub Q Mohyuddin A Helgason A Mazhar K Mansoor A Zerjal T Tyler-Smith C Mehdi SQ Y-chromosomal DNA variation in Pakistan Am J Hum Genet 2002 70 1107 1124 11898125 10.1086/339929 PMC447589

[B48] Nei M Molecular Evolutionary Genetics 1987 New York: Columbia University Press

[B49] Zhivotovsky LA Estimating divergence time with the Use of microsatellite genetic distances: impacts of population growth and gene flow Mol Biol Evol 2001 18 700 709 11319254 10.1093/oxfordjournals.molbev.a003852

[B50] Zhivotovsky LA Underhill PA Cinnio?lu C Kayser M Morar B Kivisild T Scozzari R Cruciani F Destro-Bisol G Spedini G Chambers GK Herrera RJ Yong KK Gresham D Tournev I Feldman MW Kalaydjieva L The effective mutation rate at Y chromosome short tandem repeats, with application to human population-divergence time Am J Hum Genet 2004 74 50 61 14691732 10.1086/380911 PMC1181912

[B51] Sengupta S Zhivotovsky LA King R Mehdi SQ Edmonds CA Chow CE Lin AA Mitra M Sil SK Ramesh A Usha Rani MV Thakur CM Cavalli-Sforza LL Majumder PP Underhill PA Polarity and temporality of high-resolution y-chromosome distributions in India identify both indigenous and exogenous expansions and reveal minor genetic influence of central asian pastoralists Am J Hum Genet 2006 78 202 221 16400607 10.1086/499411 PMC1380230

[B52] Bertorelle G Excoffier L Inferring admixture proportions from molecular data Mol Biol Evol 1998 15 1298 1311 9787436 10.1093/oxfordjournals.molbev.a025858

[B53] Dupanloup I Bertorelle G Inferring admixture proportions from molecular data: extension to any number of parental populations Mol Biol Evol 2001 18 4 672 675 11264419 10.1093/oxfordjournals.molbev.a003847

[B54] Maca-Meyer N Arnay M Rando JC Flores C Gonzalez AM Cabrera VM Larruga JM Ancient mtDNA analysis and the origin of the Guanches Eur J Hum Genet 2004 12 155 162 14508507 10.1038/sj.ejhg.5201075

[B55] Schneider S Roessli D Excoffier L Arlequin Version 2.000: A Software for Population Genetics Data Analysis 2000 Geneva: University of Geneva, Genetics and Biochemistry laboratory

[B56] Addinsoft: XLSTAT Software Version 7.5.2. 2008 2008 http://www.xlstat.com/

[B57] Bandelt HJ Forster P Rohl A Median-joining networks for inferring intraspecific phylogenies Mol Biol Evol 1999 16 37 48 10331250 10.1093/oxfordjournals.molbev.a026036

[B58] Delfin FC Madrid BJ Tan MP De Ungria MC Y-STR analysis for detection and objective confirmation of child sexual abuse Int J Legal Med 2005 119 158 163 15565297 10.1007/s00414-004-0503-y

[B59] Dupuy B Stenersen M Egeland T Olaisen B Y-chromosomal microsatellite mutation rates: differences in mutation rate between and within loci Hum Mutat 2004 23 117 124 14722915 10.1002/humu.10294

[B60] Gusmao L Sanchez-Diz P Calafell F Martin P Alonso CA Alvarez-Fernandez F Alves C Borjas-Fajardo L Bozzo WR Bravo ML Builes JJ Capilla J Carvalho M Castillo C Catanesi CI Corach D Di Lonardo AM Espinheira R Fagundes De Carvalho E Farfan MJ Figueiredo HP Gomes I Lojo MM Marino M Pinheiro MF Pontes ML Prieto V Ramos-Luis E Riancho JA Souza Goes AC Mutation rates at Y chromosome specific microsatellites Hum Mutat 2005 26 520 528 16220553 10.1002/humu.20254

[B61] Kayser M Brauer S Weiss G Underhill PA Roewer L Schiefenhovel W Stoneking M Melanesian origin of Polynesian Y chromosomes Curr Biol 2000 10 1237 1246 11069104 10.1016/s0960-9822(00)00734-x

[B62] Ko AM-S Chen C-Y Fu Q Delfin F Li M Chiu H-L Stoneking M Ko Y-C Early Austronesians: into and Out of Taiwan Am J Hum Genet 2014 94 426 436 24607387 10.1016/j.ajhg.2014.02.003 PMC3951936

[B63] ISOGG International Society of Genetic Genealogy (2012). Y-DNA Haplogroup Tree 2012 2012 Version: 7.45 Date: 9 August 2012, http://www.isogg.org/tree/index.html

[B64] Nonaka I Minaguchi K Takezaki N Y-chromosomal binary haplogroups in the Japanese population and their relationship to 16 Y-STR polymorphisms Ann Hum Genet 2007 71 480 495 17274803 10.1111/j.1469-1809.2006.00343.x

[B65] van Oven M Hammerle JM van Schoor M Kushnick G Pennekamp P Zega I Lao O Brown L Kennerknecht I Kayser M Unexpected island effects at an extreme: reduced Y chromosome and mitochondrial DNA diversity in Nias Mol Biol Evol 2011 28 1349 1361 21059792 10.1093/molbev/msq300

[B66] Lansing JS Cox MP de Vet TA Downey SS Hallmark B Sudoyo H An ongoing Austronesian expansion in Island Southeast Asia J Anthropol Archaeol 2011 30 262 272

[B67] Kayser M Choi Y van Oven M Mona S Brauer S Trent RJ Suarkia D Schiefenhovel W Stoneking M The impact of the Austronesian expansion: evidence from mtDNA and Y chromosome diversity in the Admiralty Islands of Melanesia Mol Biol Evol 2008 25 1362 1374 18390477 10.1093/molbev/msn078

[B68] Tofanelli S Bertoncini S Castri L Luiselli D Calafell F Donati G Paoli G On the origins and admixture of Malagasy: new evidence from high-resolution analyses of paternal and maternal lineages Mol Biol Evol 2009 26 2109 2124 19535740 10.1093/molbev/msp120

[B69] Mirabal S Cadenas A Garcia-Bertrand R Herrera R Ascertaining the role of Taiwan as a source for the Austronesian expansion Am J Phys Anthropol 2013 150 551 564 23440864 10.1002/ajpa.22226

[B70] Hammer MF Karafet TM Park H Omoto K Harihara S Stoneking M Horai S Dual origins of the Japanese: common ground for hunter-gatherer and farmer Y chromosomes J Hum Genet 2006 51 47 58 16328082 10.1007/s10038-005-0322-0

[B71] Jin HJ Kwak KD Hammer MF Nakahori Y Shinka T Lee JW Jin F Jia X Tyler-Smith C Kim W Y-chromosomal DNA haplogroups and their implications for the dual origins of the Koreans Hum Genet 2003 114 27 35 14505036 10.1007/s00439-003-1019-0

[B72] Friedlaender JS Friedlaender FR Hodgson JA Stoltz M Koki G Horvat G Zhadanov S Schurr TG Merriwether DA Melanesian mtDNA complexity PLoS One 2007 2 2 e248 *wwwplosoneorg* 2007:e248 17327912 10.1371/journal.pone.0000248 PMC1803017

[B73] Rosenberg NA Pritchard JK Weber JL Cann HM Kidd KK Zhivotovsky LA Feldman MW Genetic structure of human populations Science 2002 298 2381 2385 12493913 10.1126/science.1078311

[B74] Sanchez-Mazas A Fernandez-Viña M Middleton D Hollenbach JA Buhler S Di D Rajalingam R Dugoujon JM Mack S Thorsby E Immunogenetics as a tool in anthropological studies Immunology 2011 133 143 164 21480890 10.1111/j.1365-2567.2011.03438.x PMC3088978

[B75] Sanchez-Mazas A Osipova L Dugoujon JM Sagart L Poloni ES Sanchez-Mazas A, Blench R, Ross M, Peiros I, Lin M The GM genetic polymorphism in Taiwan aborigines: new data revealing remarkable differentiation patterns Past Human Migrations in East Asia: Matching Archaeology, Linguistics and Genetics 2008 London, New York: Routledge 313 333

[B76] Park MJ Lee HY Yang WI Shin KJ Understanding the Y chromosome variation in Korea–relevance of combined haplogroup and haplotype analyses Int J Legal Med 2012 126 589 599 22569803 10.1007/s00414-012-0703-9

[B77] Wang C-C Yan S Qin Z-D Lu Y Ding Q-L Wei L-H Li S-L Yang Y-J Jin L Li H Late Neolithic expansion of ancient Chinese revealed by Y chromosome haplogroup O3a1c-002611 Int J Syst Evol 2013 51 280 286

[B78] Mirabal S Herrera KJ Gayden T Regueiro M Underhill PA Garcia-Bertrand RL Herrera RJ Increased Y-chromosome resolution of haplogroup O suggests genetic ties between the Ami aborigines of Taiwan and the Polynesian Islands of Samoa and Tonga Gene 2012 492 339 348 22079672 10.1016/j.gene.2011.10.042

[B79] Pierson MJ Martinez-Arias R Holland BR Gemmell NJ Hurles ME Penny D Deciphering past human population movements in Oceania: provably optimal trees of 127 mtDNA genomes Mol Biol Evol 2006 23 1966 1975 16855009 10.1093/molbev/msl063 PMC2674580

[B80] Mona S Grunz KE Brauer S Pakendorf B Castri L Sudoyo H Marzuki S Barnes RH Schmidtke J Stoneking M Kayser M Genetic admixture history of Eastern Indonesia as revealed by Y-chromosome and mitochondrial DNA analysis Mol Biol Evol 2009 26 1865 1877 19414523 10.1093/molbev/msp097

[B81] Delfin F Myles S Choi Y Hughes D Illek R van Oven M Pakendorf B Kayser M Stoneking M Bridging near and remote Oceania: mtDNA and NRY variation in the Solomon Islands Mol Biol Evol 2012 29 545 564 21771715 10.1093/molbev/msr186

[B82] Solheim WG Archaeology and Culture in Southeast Asia: Unraveling the Nusantao 2006 Diliman, Quezon City: University of the Philippines Press 316 ISBN 971-542-508-9

[B83] Tsang CH The prehistory of Taiwan: a brief introduction Seventeenth Congress of the Indo-Pacific Prehistory Association; Sep 9–15 2002 Taipei (Taiwan): Academia Sinica 47 52

[B84] Zhong H Shi H Qi XB Duan ZY Tan PP Jin L Su B Ma RZ Extended Y chromosome investigation suggests postglacial migrations of modern humans into East Asia via the northern route Mol Biol Evol 2011 28 717 727 20837606 10.1093/molbev/msq247

[B85] Sharma S Rai E Bhat AK Bhanwer AS Bamezai RN A novel subgroup Q5 of human Y-chromosomal haplogroup Q in India BMC Evol Biol 2007 7 232 18021436 10.1186/1471-2148-7-232 PMC2258157

[B86] Razafindrazaka H Ricaut F Cox M Mormina M Dugoujon J Randriamarolaza L Guitard E Tonasso L Ludes B Crubezy E Complete mitochondrial DNA sequences provide new insights into the Polynesian motif and the peopling of Madagascar Eur J Hum Genet 2010 18 575 581 20029456 10.1038/ejhg.2009.222 PMC2987306

[B87] Abdulla MA Ahmed I Assawamakin A Bhak J Brahmachari SK Calacal GC Chaurasia A Chen CH Chen J Chen YT Chu J la Paz EM C-d De Ungria MC Delfin FC Edo J Fuchareon S Ghang H Gojobori T Han J Ho SF Hoh BP Huang W Inoko H Jha P Jinam TA Jin L Jung J Kangwanpong D Kampuansai J Kennedy GC Mapping human genetic diversity in Asia Science 2009 326 1541 1545 20007900 10.1126/science.1177074

