# Supplementary material for: Taiwan Y-chromosomal DNA variation and its relationship with Island Southeast Asia
Source: BMC Genet. 2014 Jun 26;15:77. doi: 10.1186/1471-2156-15-77 (PMC4083334; doi:10.1186/1471-2156-15-77)
Supplement: Additional file 1: Figure S1 — Frequency distributions of O clades (O1, O2 and O3) in samples from Taiwan and two neighboring populations (Fujian Han, and Ivatan from Batan). Figure S2. Frequency distributions of O clades (O1, O2 and O3) in pooled samples from Taiwan compared to the Philippines, Indonesia and mainland Southeast Asia. Figure S3. Variation of diversity measures according to latitude in TwMtA. The three plots display the estimated values and linear regression of, respectively, gene diversity (Additional file 1: Table S2), frequency of haplogroup O1a1*-P203 (Additional file 1: Table S2), and STR diversity of haplogroup O1a1*-P203 (measured by the rho statistic, Additional file 1: Table S3), on latitude. Pearson’s linear correlation coefficient and its statistical significance are given in the respective three captions. Figure S4. MDS plot (stress 0.203) using our data and literature data from Additional file 1: Table S1 (over 6000 chromosomes). Haplogroup frequencies were adjusted to 20 basal haplogroups (low definition SNP). See Additional file 1: Table S1 for correspondence of numbers and populations. Table S1. Asian population data from this study and from previously published studies. Data used for MDS analysis shown in Additional file 1: Figure S3. Table S2. Frequency distributions and gene diversity of Y-SNP haplogroups in populations from Taiwan, Island Southeast Asia and Indochina. Table S3. Age (in 1000 years) and Standard Error (SE) of Y-STR variation within Haplogroups (7 STRs). Table S4. Y-SNP and Y-STR raw data. [file 1471-2156-15-77-S1.zip › additional file/1860087297111575_Figure S2.pdf]

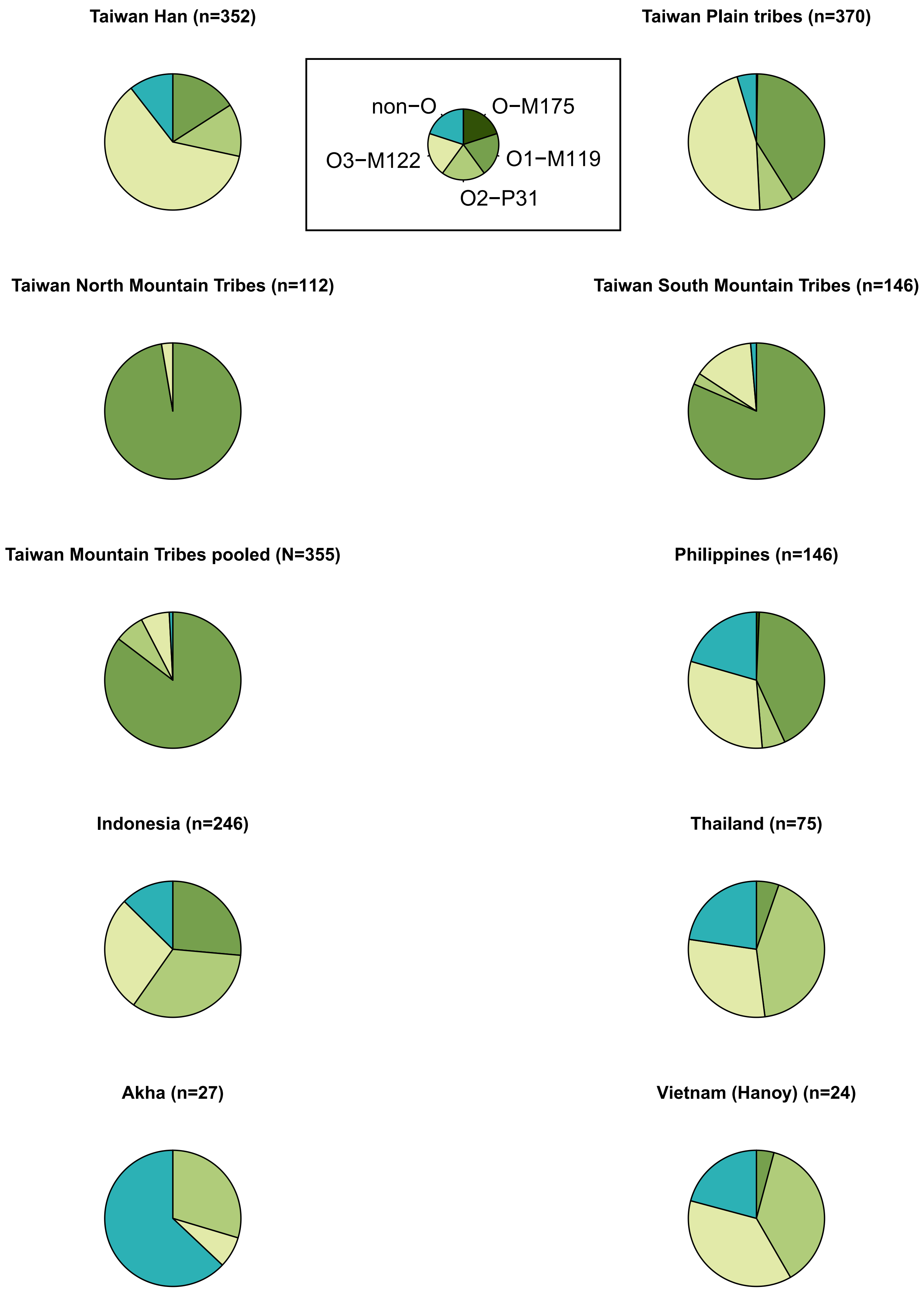
Taiwan Han (n=352)
Taiwan Plain tribes (n=370)
non−O
O−M175
O3−M122
O1−M119
O2−P31
Taiwan North Mountain Tribes (n=112)
Taiwan South Mountain Tribes (n=146)
Taiwan Mountain Tribes pooled (N=355)
Philippines (n=146)
Indonesia (n=246)
Thailand (n=75)
Akha (n=27)
Vietnam (Hanoy) (n=24)
